# Pre- and post-operative administration of omega-3 polyunsaturated fatty acids in cardiac surgery patients. A narrative review

**DOI:** 10.1097/MS9.0000000000003061

**Published:** 2025-03-18

**Authors:** Athanasios Athanasiou, Marinos Charalambous, Theodora Anastasiou, Elpidoforos S. Soteriades

**Affiliations:** a Department of Cardiothoracic Surgery, Nicosia General Hospital, Nicosia, Cyprus; b Heart and Vascular Institute, University of Pittsburgh Medical Center, Pittsburgh, Pennsylvania, USA; c Department of Medicine, University of Cyprus, Nicosia, Cyprus; d Department of Environmental Health, Environmental and Occupational Medicine and Epidemiology, Harvard T.H. Chan School of Public Health, Boston, Massachusetts, USA; e Healthcare Management Program, School of Economics and Management, Open University of Cyprus, Nicosia, Cyprus

**Keywords:** cardiac surgery, N-3 PUFA, omega-3 fatty acids, postoperative, preoperative

## Abstract

Eicosapentaenoic acid (EPA), and docosahexaenoic acid (DHA) are two biologically active omega-3 polyunsaturated fatty acids (n-3 PUFA), acquired by nutrition and incorporated in cell membranes’ phospholipids, thus playing a crucial role in human health and homeostasis. Due to their potential cardioprotective, anti-inflammatory, and anti-arrhythmic actions, n-3 PUFA emerge as an interesting therapeutic option for cardiac surgery (CS) patients. The aim of this review was to assess the effects of perioperative administration of n-3 PUFA in CS patients. A comprehensive literature search was conducted in order to identify prospective cohort studies and randomized controlled trials (RCT) reporting on the perioperative effects of n-3 PUFA among adult patients undergoing CS. A total of 31 articles, published between 1995 and 2022, including 10 543 patients, met the inclusion criteria. There seems to be a beneficial effect of n-3 PUFA supplementation for arrhythmias such as in Postoperative Atrial Fibrillation (POAF), reduction of Intensive Care Unit Length of Stay (ICULOS) & Hospital Length of Stay (HLOS), reduction in postoperative ventilation time, in inotropic demand, in postoperative fatigue, as well as in overall morbidity and mortality. Moreover, n-3 PUFA increase antioxidant potential, attenuate oxidative stress and inflammation with subsequent significant reduction in myocardial ischemia/reperfusion (I/R) injury, thus promoting early metabolic recovery of the heart after elective CS leading to improved myocardial protection. They represent a readily available and cost-effective strategy that could improve the outcome of patients undergoing CS, by reducing the risks of serious cardiovascular adverse events (AE), both peri- and post-operatively.

## Introduction

In recent years, a growing body of research has focused on the role of omega-3 (n-3) fatty acids, a family of biologically active polyunsaturated fatty acids (PUFA), in human health and homeostasis (Tables 1 and 2). N-3 PUFA are present in cell membranes and are incorporated primarily into phospholipids^[1]^. They cannot be synthesized by mammals and thus must be obtained by diet, therefore considered essential fatty acids. They are found primarily in fatty fish, plant sources or fish oil supplements^[2,3]^. Essential n-3 PUFA include plant-derived a-linolenic acid (ALA), fish-oil-derived stearidonic acid (SDA), docosapentaenoic acid (DPA), eicosapentaenoic acid (EPA), and docosahexaenoic acid (DHA)^[1]^. Since the majority of research has turned its focus on EPA and DHA, we too emphasize on these n-3 PUFA in this review. The European Food Safety Authority (EFSA) makes recommendations for combined EPA and DHA intake^[4]^ for 250 mg of EPA/DHA per day for adults and children over the age of 2 while in the United Kingdom (UK) long-chain omega-3 fatty acid intake has been recommended to increase from 200 to 450 g/day; equating to one portion of white and one portion of oily fish per week^[5]^. The US Food and Drug Administration (FDA) classifies n-3 PUFA as generally recognized as safe (GRAS) and recommends that a daily intake not exceed 3 g/day of EPA and DHA combined, with no more than 2 g/day deriving from supplements^[6]^. The American Heart Association (AHA) recommends eating at least 2 meals of oily fish per week^[7]^ which corresponds to 1 g of EPA + DHA per day. To patients with an increased risk of cardiovascular disease, they suggest the additional consumption of 600 mg EPA + DHA per day, and to those with a family history of sudden death, an additional 2 g/day^[8]^. Statements published by AHA^[9,10]^ recommend the prescription of 2–4 g of polyunsaturated fatty acids (EPA und DHA) to lower elevated plasma triglyceride levels to a range under 200 mg/dL^[11]^, as well as have a beneficial effect on elevated plasma low-density lipoprotein (LDL) levels.^[12-14]^ N-3 PUFA and their metabolites act via multiple mechanisms, and modulate a number of important physiologic responses^[1,15]^. Their role is being investigated against various chronic degenerative diseases, such as cardiovascular disease^[16]^, rheumatoid arthritis^[17]^, inflammatory bowel disease^[18]^, depression^[19]^, and cancer^[20]^. In this context, the administration of n-3 PUFA as a pharmaco-nutrient strategy represents a promising and attractive therapeutic option for a variety of diseases and interventions. An extensive amount of evidence suggests that n-3 PUFA have multiple cardioprotective properties in both primary and secondary coronary heart disease (CHD).^[10,14,21-30]^

**HIGHLIGHTS**

- This narrative review assesses the effects of perioperative supplementation of n-3 PUFA, namely eicosapentaenoic acid (EPA), and docosahexaenoic acid (DHA), for CS patients by reviewing 31 cohort studies and randomized controlled trials.
- N-3 PUFA exhibit broad cardioprotective effects, influencing various parameters in coronary heart disease pathogenesis, including inflammation, triglyceride formation, blood pressure, platelet aggregation, and vascular relaxation.
- Postoperative levels of n-3 PUFA may decrease in certain group of patients, leading to potentially cardiovascular risks if not adequately substituted.
- Evidence supports the effectiveness of peri/postoperative n-3 PUFA supplementation in reducing postoperative arrhythmias, shortening ICU and hospital stay, and lowering overall cardiovascular disease risk and mortality.
- N-3 PUFA postoperative administration is considered safe, and some researchers advocate for its routine use in post-CABG therapy as a cost-effective strategy.

**Table 1:** Summary of n-3 PUFA influence on different systems.

System	Effects of n-3 PUFA
Cardiovascular	Reduce cardiovascular morbidity and mortality^[94]^ including sudden cardiac death.^[95-98]^
	Improve heart failure outcomes^[99]^.
	Reduce triglycerides^[100]^ and VLDL^[101]^, blood pressure, platelet aggregation, arrhythmia, and atherogenesis^[102]^.
	Interrupt of vascular thrombus formation and vascular lesion formation^[81]^
	Regulate blood pressure^[103]^.
	Stable atherosclerotic plaques^[104]^.
	Prevent significant coronary events in hypercholesterolemic patients^[14]^.
	Lower the risk for atrial fibrillation^[105]^.
	Reduce the risk of fatal ventricular arrhythmias^[106]^.
	Reduce vascular wall thickness^[107]^.
	Protect against endothelial dysfunction^[108]^.
	Prevent postoperative acute lung injury^[109]^.
	Reduce the risk for postoperative atrial fibrillation.^[38-40]^
	Reduce the rate of repeat revascularization^[63]^.
	Reduce intensive care unit (ICU) & hospital length of stay^[83]^.
	Reduce intimal hyperplasia in autologous vein grafts reducing the incidence of vein graft occlusion^[46,110,111]^.
	Control fatigue after CABG^[78]^.
Gastrointestinal	Reduce triacylglycerol production^[95]^.
	Protect against pancreatic beta-cell damage and diabetes^[112]^.
	Lower risk of hepatic steatosis^[113]^ and nonalcoholic fatty liver disease^[114]^.
Immune	Improve the balance between NO and harmful reactive oxygen radicals (ROS)^[32]^.
	Reduce systemic inflammatory response and prevent multiple organ dysfunction^[33]^.
	Contribute to inflammation resolution^[115,116]^.
	Increase the expression of genes for antioxidant enzymes^[117]^.
	Alleviate symptoms of rheumatoid arthritis, such as pain and stiffness^[118]^.
	Protect against Crohn’s disease and inflammatory bowel disorders^[35]^.
Musculoskeletal	Protect against osteoporosis by maintaining bone mass^[94]^, especially in postmenopausal women^[119]^.
Nervous	Lower risk for Alzheimer’s disease and cognitive decline^[120,121]^.
	Aid in managing multiple sclerosis^[122]^.
	Regulate synaptic plasticity^[123]^, neurotransmission, cell survival and neuroinflammation^[124]^.
	Enhance growth and neuronal development, and immune function in infants^[125,126]^.
	Support fetal and infant brain and visual system development.^[127]^
	Lower risk for depression^[128,129]^.
	Enhance concentration and prevent ADHD^[130]^.
	Improve autism characteristics including stereotyped behaviors and social communication^[131]^.
	Relieves anxiety in individuals without an anxiety disorder^[132]^.
Renal	Supplementary treatment for IgA nephropathy^[133]^.
Reproductive	Alleviate premenstrual syndrome symptoms^[134]^.
	Enhance fertility in women^[135]^ including those with polycystic ovary syndrome^[136]^.
	Reduce hot flashes and quality of life in menopausal women^[137]^.
	Promote placental growth through angiogenesis in trophoblast cells^[138]^.
Respiratory	Reduce the incidence of infectious respiratory diseases in children^[127,139]^.
	Provide antibacterial effects and help resolve inflammation in Mycobacterium tuberculosis infection^[115]^.
	Benefit chronic airway inflammatory diseases such as chronic obstructive pulmonary disease^[140]^.
Skin	Possesses anti-aging effects by increasing collagen and elastic fiber gene expression^[141]^.
	Supplementary treatment of chronic inflammatory skin diseases, such as atopic dermatitis, psoriasis, and acne^[142]^.
Visual	Lower risk of diabetic retinopathy^[143]^.
	May help manage myopia^[144]^.
Cancer	Regulate tumor cell growth^[94]^.
	Protect various types of cancer including prostate, colon, breast^[145]^, lung, colorectal, ovarian, pancreatic, skin, and stomach^[146,147]^.
	Improve the efficacy and tolerability of chemotherapy^[148]^.
	Inhibit bone reabsorption in patients taking aromatase inhibitors^[149]^.
	Lower breast cancer recurrence and improved overall mortality^[145]^.

**Table 2:** Consequences of n-3 PUFA deficiency on different systems.

System	Effects of n-3 PUFA deficiency
Cardiovascular	Increases the risk for cardiovascular events^[151]^.
	Accelerates the progression of coronary artery disease^[30]^.
	Accelerates the process of endothelial dysfunction^[152]^.
	Increases the presence and severity of coronary plaques^[104]^.
	Increases the risk for peripheral artery disease^[153]^.
Gastrointestinal	Increases the risk for non-alcoholic fatty liver disease^[152]^.
	Alters gut microbiota leading to dysbiosis^[150]^.
	Aggravates streptozotocin-induced pancreas injury^[154]^.
	Predisposes individuals to metabolic syndrome^[155]^.
Immune	Aggravates inflammatory status and oxidative stress^[116]^.
	Increases risk for breast cancer^[145]^.
Nervous	Disrupts brain functions by interfering with oligodendrocyte maturation and myelination during neurodevelopment^[156]^.
	Disrupts multiple neurotransmission systems, including the dopaminergic and serotonergic systems^[112]^.
	Promotes progressive neuropathological processes.^[157-159]^
	Impairs attention, learning, and memory performance^[160]^.
	Increases inflammation-induced spatial memory impairment^[161]^.
	Increases risk for age-related cognitive decline^[162]^.
	Changes genes associated with neuronal plasticity and the brain’s innate immune system^[163]^.
	Heightens the risk of autism spectrum disorder, attention deficit hyperactivity disorder, and schizophrenia^[123]^.
Reproductive	Increases susceptibility to testicular dysfunction, affecting both germ cell supply and hormone secretion^[163]^.

Additionally, there is growing clinical evidence that n-3 PUFA have beneficial effects on acute and chronic inflammatory diseases, by blocking inflammatory pathways^[1,31]^. They enhance production of the vasodilator nitric oxide (NO) by the endothelium and improve the balance between NO and harmful reactive oxygen radicals (ROS)^[32]^. Therefore, n-3 PUFA reduce systemic inflammatory response and tissue or organ dysfunction^[33]^.

There are several clinical studies reporting that n-3 PUFA act as immunomodulators. They regulate T-cell differentiation and inflammatory autoimmune response by reducing antigen presentation through major histocompatibility complex II (MHC II)^[34]^. They also exert an inhibitory effect on the activation of immune cells in both endogenous (from birth) and acquired immunity (e.g., vaccines)^[35]^. Additionally, there are studies supporting that they modulate gut microbiota by decreasing Firmicutes and increasing Bacteroidetes which lower the Firmicute/Bacteroidete ratio. Increases in the Firmicute/Bacteroidete ratio have been connected to a variety of metabolic disorders. For these reason, n-3 PUFA are considered as “candidate prebiotics”^[36,37]^.

Due to their proposed cardioprotective, anti-inflammatory, and anti-arrhythmic actions, n-3 PUFA emerge as a promising therapeutic option for cardiac surgery (CS) patients. Systemic inflammation due to ischemia-reperfusion, oxidative stress, and blood contact with non-endothelial surfaces during extra-corporeal circulation, are key features in patients after CS. These important clinical implications have been advocated by several studies in order to identify the role of n-3 PUFA in the prevention of common postoperative complications and arrhythmias e.g., postoperative atrial fibrillation (POAF).^[38-40]^

Overall, a better comprehension of n-3 PUFA’ implications in CS, is needed, and therefore the purpose of this paper was to evaluate the current peri- and post-operative use of n-3 PUFA in adult CS patients, namely the clinical settings, dosage and duration, clinical outcomes and adverse effects.

## Materials and methods

A comprehensive literature search was conducted in four major electronic databases (Medline/PubMed, Cochrane Library, Embase/Elsevier and Google Scholar) in order to identify prospective cohort studies and/or randomized controlled trials (RCTs) reporting on the pre- and post-operative administration of n-3 PUFA among CS patients. The search covered the period between January 1980 and December 2023.

Predefined search terms included “n-3 PUFA,” “Omega-3 fatty acids,” “polyunsaturated fatty acids” and “cardiac surgery,” “cardiothoracic surgery,” “heart surgery,” “cardiopulmonary bypass,” “CPB,” “extracorporeal circulation,” “ECC,” “coronary artery bypass grafting,” “CABG,” “CAB,” “valve surgery” and “valvular surgery.” There was no language limitation. Studies that were included into the analysis met the following criteria: (i) prospective cohort studies and/or RCTs, (ii) adult patients (>18 years old) undergoing CS, (iii) comparison of n-3 PUFA with a control or placebo group, (iv) studies that reported data on the pre- and/or post-operative administration of n-3 PUFA (duration, dosage), and (v) studies that reported information on the incidence of postoperative complications and/or benefits following administration of n-3 PUFA in adult CS patients.

**Figure 1.**
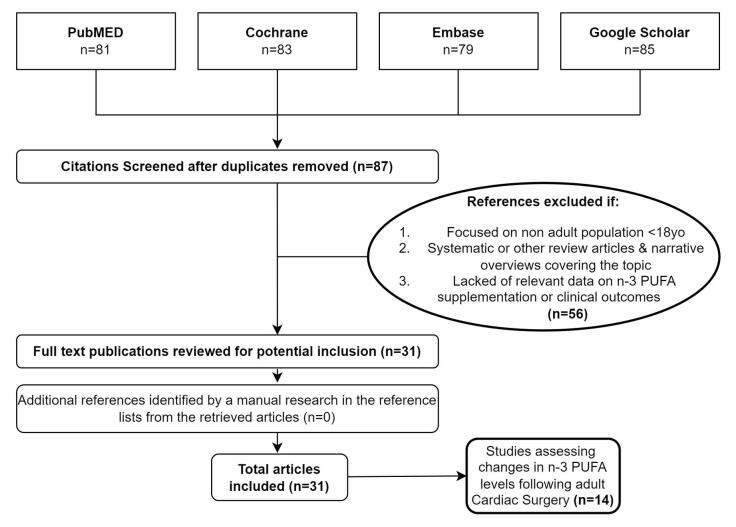
Flow chart with a graphical representation of the literature selection process for the present article.

Studies were excluded if they reported on non-adult patient populations (<18 years old). Review articles focusing on the topic were excluded, however, the reference list of all review articles identified, were searched in order to include any additional original articles that met the inclusion criteria and were not identified through the extensive databases’ search. A flow chart with the literature selection process is shown in Fig. 1

Implemented statistical analysis^[41,42]^ (mean comparison t-test, analysis of variance – ANOVA, and graphical representation with clustered boxplots), was carried out with IBM SPSS^®^ 20 software^[43]^ and the chosen level of statistical significance was 5% (*P* = 0.05)^[41]^.

## Results

In brief, a total of 87 published articles were identified through the initial literature search from the four major electronic databases. Following abstract review, we excluded 56 manuscripts that did not include clinical events based on the principal inclusion and exclusion criteria. The 31 remaining articles, with a total population of 10 543 patients, were examined in full-text and included in the present narrative review since they met our inclusion criteria (Fig. 1). All enrolled studies reported on n-3 PUFA administration regimens with 4991 patients receiving treatment, solely or amongst with other antioxidants or supplements; as well as on the pre- and post-operative effects among CS patients: namely the clinical setting, dosages, duration, potential complications or benefits and the primary or secondary clinical outcomes. A detailed list of the enrolled studies is presented in Table 3 with their characteristics and outcome measures presented in Table 5.

**Table 3:** Studies included in the review. Characteristics, inclusion and exclusion criteria.

Author, type of study & operation, study groups, mean age (years), male sex (*♂*)	Inclusion criteria (IC)
	Exclusion criteria (EC)
**Calo**^[65]^, RCT – No blinding, **OP**: CABG | **CPB**: 141/160, PUFA: 79, Ctrl: 81, **Sum**: 160, **Mean age**: 65.6 ± 8.5, ***♂*:** 136/160	**IC**: elective CABG, >18 years old, SR, stable hemodynamic conditions.
	**EC**: concomitant valvular surgery, history of supraventricular arrhythmias, use of AAD (other than beta-antagonists, calcium-channel antagonists, or digitalis).
**Saravanan**^[50]^, RCT – DB, **OP**: CABG | **CPB**:100%, PUFA: 52, Ctrl: 51, **Sum**:103, **Mean age**: PUFA: 64 | Control: 68**, *♂*:** 82/103	**IC:** >18 years, elective isolated CABG on CPB.
	**EC**: history of arrhythmia, AAD (class 1 or 3), fish oil supplements taken within the previous 3 m.
**Heidt**^[11]^, RCT – DB, **OP**: CABG | **CPB**: 100%, PUFA: 52, Ctrl: 50, **Sum**: 102, **Mean age**: 67 ± 9.3**, *♂*:** 70/102	**IC:** >18 years old, SR, stable hemodynamic conditions, without angina at rest.
	**EC**: concomitant valve surgery, history of supraventricular arrhythmias, use of AAD (other than beta-blockers and calcium channel antagonists)
**Heidarsdottir**^[47]^, RCT – DB, **OP**: CS | **CPB**: 100%, PUFA: 83, Ctrl: 85, **Sum**: 168, **Mean age**: 67**, *♂*:** 79.2%	**IC**: CABG or valve surgery.
	**EC:** <40 years old, history of supraventricular arrhythmias, AAD (amiodarone ± sotalol), emergent operation
**Sandesara**^[51]^, RCT – DB, **OP: CS | CPB**: 182/243, PUFA: 120, Ctrl (n6-PUFA):123, **Sum**: 243, **Mean age**: 62.7, *♂*: 81%	**IC**: patients 18–85 years old, elective CABG ± valve surgery.
	**EC**: emergency CABG, unstable angina or HF, history of AF, planned Maze procedure or pulmonary vein isolation, warfarin administration 48 h preop, use of Vaughan-Williams Class I or III AAD within 5 elimination half-lives of the drug (or within 2 months for amiodarone), pacemaker or cardioverter-defibrillator, n3-PUFA supplementation at the time of screening, pregnancy, inability to provide consent.
**Farquharson**^[48]^, RCT – DB, **OP**: CS | **CPB**: 193/194, PUFA: 97, Ctrl: 97, **Sum**: 194, **Mean age**: PUFA: 64 ± 11 | Ctrl: 64 ± 10**, *♂*:** 142/194	**IC:** >18 years old undergoing CS
	**EC**: AF or atrial flutter, AAD class 1 or 3 within the previous 3 m, urgent surgery (<3 weeks), NYHA stage IV, MI within the previous 2 weeks, any condition that might affect the ability to ingest or absorb dietary fat, patients who consumed dietary supplements rich in n-3 oils, e.g., fish oil or flaxseed oil, or self-reported habitual consumption of ≥1 fish meal per week.
**Veljović**^[82]^, RCT – ND, **OP**: CABG | **CPB**: 100%, PUFA: 20, Ctrl: 20, **Sum**: 40, **Mean age: PUFA:** 65.3 ± 8 | Ctrl: 62.4 ± 7, *♂*: 35/40	**IC**: elective on-pump CABG, >18 years old, SR, stable hemodynamic conditions preop.
	**EC**: emergency or redo CABG, combined other cardiac procedure, Q-wave MI in the last 6 weeks, unstable angina, poor left ventricular function, abnormal coagulation tests, coagulopathy, preop treatment with other anticoagulants.
**Sorice**^[66]^, RCT – No blinding, **OP**: CABG | **CPB**: 108/201, PUFA: 96, Ctrl: 105, **Sum**: 201, **Mean age**: 63 ± 10**, *♂*:** 164/201	**IC:** >18 years, normal SR, stable hemodynamic condition preop.
	**EC**: history of AF, AAD (unless with beta-blockers, Ca-antagonists), severe valvular disease requiring surgery, different technical strategy of the surgeon.
**Farahani**^[67]^, **RCT – DB, OP**: CABG | **CPB**: 372/401, PUFA: 202, Ctrl: 199 **Sum**: 401, **Mean age**: PUFA: 60.62 ± 0.63 Ctrl: 61.28 ± 0.71, *♂*: 259/401	**IC**: elective CABG, >18 years old, willing to participate, hemodynamically stable, SR.
	**EC**: received fish oil supplements in the past 90 d, AAD class I ± class III, history of supraventricular arrhythmias, MI within 2 weeks, emergent operation, concomitant valvular surgery, medical conditions affecting fat absorption, patients’ inability to comply, pacemaker implantation postop.
**Gholami**^[78]^, RCT – TB, **OP**: CABG | **CPB**: 100%, PUFA: 80, Ctrl: 80, **Sum**: 160, **Mean age**: PUFA: 61.41 ± 10.51 | Ctrl: 63.93 ± 9.97, ***♂***: 82/160	**IC**: ≥ 18 years old, no prior use of antioxidants, no use of fish oil over the past 3 months, lack of diseases with high inflammatory components.
	**EC**: hypersensitivity to n-3 PUFA or Vit C, heart attack during or following surgery.
**Rodrigo**^[61]^, RCT – DB, **OP**: CS | CPB: 100%, PUFA: 103, Ctrl: 100, **Sum** = 203, **Mean age**: N-3 PUFA: 61| Control: 58.5, ***♂*:** 173/203	**IC:** ≥ 18 years old, scheduled CABG ± valve surgery, SR.
	**EC**: history of arrhythmia or MI, use of amiodarone or sotalol, severe CHF (NYHA III or IV), prosthetic valves, congenital valvular disease, left atrial diameter >50 mm, conditions associated with oxidative stress or inflammation such as chronic rheumatic or neoplastic diseases, liver insufficiency, severe chronic kidney disease (creatinine >2.0 mg/dL), recent infections, use of NSAID, corticosteroids, antioxidant vitamins, or fish oil supplements 3 m preop.
**Veljović**^[80]^, RCT – ND, **OP**: CABG | **CPB**: 100%, PUFA: 20, Ctrl: 20, **Sum**: 40, **Mean age**: PUFA: 65.3 ± 8 | Ctrl: 62.4 ± 7, *♂*: 35/40	**IC**: first on-pump CABG surgery, >18 years old, SR, stable hemodynamic conditions preop.
	**EC**: emergency CABG, redo CABG, combined CABG, Q-wave MI in the last 6 weeks, unstable angina, poor left ventricular function.
**Akintoye**^[57]^, RCT – DB (OPERA), **OP**: CS | **CPB**: ND, PUFA: 758, Ctrl: 758, **Sum**: 1516, **Mean age**: 63, *♂*: 72%	**IC**: age ≥18 years, upcoming CS, SR
	**EC**: regular use (≥3 d/week) of fish oil within the prior 4 weeks, known allergy to fish oil or olive oil, current pregnancy, inability to provide informed written consent.
**Lomivorotov**^[91]^, RCT – DB, **OP**: CABG | **CPB**: 100%, PUFA: 17, Ctrl: 21, **Sum**: 39, **Mean age**: PUFAs: 61 | Control: 58, *♂*: 37/39	**IC: >**18 years old undergo elective CABG using CPB.
	**EC**: unstable angina requiring intervention or CABG <24 h after screening, decompensated congestive HF, AF, uncorrected significant valvular heart disease, known hypersensitivity to the study drug, LVEF <35%, use of AAD other than beta-blockers, non-cardiac illness with a life expectancy <1 year, bleeding diathesis, coagulopathy, significant renal and liver insufficiency, significant thyroid or pulmonary disease, uncontrolled diabetes mellitus, pacemakers, unable to provide consent, taking marine-based omega-3 fish oil supplements, disturbances in lipid metabolism (serum triglyceride >3 mmol/L).
**Skuladottir**^[58]^, RCT – DB, **OP**: CABG (**CPB**: PUFA: 87.1% | Ctrl: 81%), PUFA: 62, Ctrl: 63, **Sum**: 125, **Mean age**: PUFA: 69| Control: 66, *♂*: PUFA: 79% | Control: 81.4%	**IC**: elective or semi-emergent CABG
	**EC**: age <40 years old, history of supraventricular arrhythmias, use AAD (amiodarone and/or sotalol).
**Castillio**^[62]^, RCT – DB, **OP**: CS | **CPB**: 100%, PUFA: 48, Ctrl: 47, **Sum** = 95, **Mean age**: 59, ***♂*:** 68/95	**IC**: elective on-pump CS, SR.
	**EC**: history of AF, congenital or previous CS, cirrhosis, chronic renal failure (creatinine >150 mmol/L), inflammatory or rheumatic diseases, use of corticosteroid, anti-inflammatory or antioxidant drugs, use fish oil supplements 3 months preop.
**Jackson**^[52]^, RCT – DB, Prosp. ancillary (OPERA Cognitive), **OP**: CS | **CPB**: ND, PUFA: 159, Ctrl: 161, **Sum**: 320, **Mean age**: 62, *♂*: 226/320 (71%)	**IC**: age ≥18 years old, scheduled CS, SR
	**EC**: regular use of fish oil, known allergy or intolerance to fish oil or olive oil (placebo), current pregnancy, existing or planned cardiac transplant or use of ventricular assist device, unable or unwilling to provide informed consent.
**Masson**^[68]^, RCT Prosp. ancillary (OPERA), **OP**: CS | **CPB**: 95.5%, PUFA: 290, Ctrl 272, **Sum**: 562, **Mean age**: 62.6 ± 12.7, *♂*: 71.9%	**IC**: age ≥ 18 years, SR, scheduled CS
	**EC**: OPERA trial
**Mariscalco**^[76]^, Prosp. observational, **OP**: CS | **CPB**: 100%, PUFA: 84, Ctrl: 446, **Sum**: 530, **Mean age**: 66.4 ± 10.9, ***♂*:** 68.5%	**IC**: elective or emergent procedures, redo operations.
	**EC**: preop chronic, presence of pacemaker.
**Feguri**^[77]^, RCT – DB, **OP:** CABG | **CPB:** 100%, PUFA: 43, Ctrl: 14, **Sum**: 57, Mean age: 63.50|63 × 63|65, *♂*: 38/57	**IC**: 18–80 years old with CAD and eligible for elective on-pump CABG.
	**EC:** ≤ 18 years old, not informed consent, insulin-dependent diabetes, fasting blood glucose level > 150 mg/dL, gastroparesis or gastroesophageal reflux disease, use of n-3 PUFA or corticosteroids >6 months preop, liver cirrhosis (Child class A, B or C), acute or chronic kidney injury (creatinine ≥2.0 mg/dL), patients on hemodialysis, uncontrolled dyslipidemia, coagulopathy ± low platelet count, allergy to fish or shrimp, emergency surgery, reoperation, combined surgical procedures, acute coronary syndrome and mechanical complications of MI, subjective global assessment class C, any type of transfusion 3 months preop.
**Stanger**^[79]^, RCT – DB, **OP**: CABG | **CPB**: ND, Group 1 (Ctrl): 20, Group 2 (Vitamins):19, Group 3 (PUFA): 19, Group 4 (Vitamins & PUFA): 17, **Sum** = 75, **Mean age**: 66 ± 8, ***♂*:** 68/75	**IC**: good pharmacological control, lipid profile, blood pressure values and glycemic control.
	**EC**: ND
**Mozaffarian**^[53]^, RCT – DB (OPERA), **OP**: CS | **CPB**: ND, PUFA: 758, Ctrl: 758, **Sum**: 1516, **Mean age**: 64 ± 13, *♂*: 72.2%	**IC:** >18 years old, scheduled CS, SR at enrollment
	**EC**: use of fish oil during the past 4 weeks, known allergy or intolerance to fish oil or olive oil, pregnancy, planned or existing cardiac transplant or left ventricular assist device, unable or unwilling to provide informed written consent.
**Gu**^[54]^, Prosp. cohort, **OP**: CS | **CPB**: 100%, PUFA: 18, Ctrl: 31, **Sum**: 49, **Mean age**: 66.0 ± 10.4, *♂*: 35/50	**IC**: first-time elective CS, SR preop
	**EC**: history of AF or atrial flutter, pacemaker, non-elective surgery, off-pump CABG, pregnancy, lactating patients
**Rodrigo**^[60]^, RCT – DB, **OP**: CS | CPB: 100%, PUFA: 77, Ctrl: 75, **Sum**: 152, Mean age: patients > 60: VitC: 66 |Control: 65. Patients <60: VitC: 53 |Control: 52, ***♂*:** 120/152	**IC**: scheduled CS with extracorporeal circulation.
	**EC**: previous CS, chronic or paroxysmal AF, history of arrhythmias, advanced pulmonary or hepatic disease, chronic renal failure (creatinine >2.0 mg/dL).
**Benedetto**^[63]^, Prosp. cohort, **OP**: CABG, **CPB**: 100%, PUFA: 930, Ctrl: 1170, **Sum**: 2100, **Mean age**: PUFA: 68 | Control: 67, ***♂*:** PUFA: 79% | Control: 83%	**IC**: adults underwent isolated CABG.
	**EC**: patients who died within 30 days from surgery, patients treated with n-3 PUFAs who stopped therapy after discharge for any reason, patients who were prescribed n-3 PUFAs late after discharge.
**Andreassen**^[45]^, RCT – DB, **OP**: | **CPB**: heart transplant, PUFA: 14, Ctrl: 14, **Sum**: 28, **Mean age**: PUFA: 29 ± 2 | Control: 33 ± 3, *♂*: PUFA: 73% |Control: 85%	**IC**: ND
	**EC**: ND
**Akintoye**^[49]^, RCT Prosp. ancillary (OPERA) – DB, **OP**: CS | **CPB**: ND, PUFA: 249, Ctrl: 230, **Sum**: 479, **Mean age**: 63, *♂*: 73%	**IC:** ≥18 years, scheduled CS, SR at enrollment.
	**EC**: regular use (≥3 days/week) of fish oil within the prior 4 weeks, known allergy to fish oil or olive oil (placebo), current pregnancy, inability to provide informed written consent.
**Charman**^[64]^, Pilot study – DB, **OP**: CABG | **CPB**: 100%, PUFA: 22, Ctrl: 18, **Sum**: 40, **Mean age**: PUFA: 62.27 ± 6.7 | Control: 59.97 ± 6.7, *♂*: 35/40	**IC**: routine CABG, free from aspirin or other non-steroidal analgesic for at least 7 days preop.
	**EC**: patients on warfarin, diabetes, dependent upon IV nitrates or heparin.
**Rubanenko**^[44]^, RCT – ND, **OP**: CABG | **CPB**: 274/306, PUFA: 148, Ctrl: 158, **Sum**: 306, **Mean age**: 62, *♂*: 263/306 (85.9%)	**IC**: scheduled CABG
	**EC**: ND
**Berger**^[56]^, RCT – DB, **OP**: CS | **CPB**: 100%, PUFA: 14, Ctrl: 14, **Sum**: 28, **Mean age**: 65.5 ± 9.9, *♂*: 25/28	**IC**: 18–85 years old, SR
	**EC**: no consent, participation in another trial, emergency, or heart-beating surgery, AAD use, uncontrolled dyslipidemia, acute or chronic renal failure (plasma creatinine concentration >150 mmol/L), liver cirrhosis (child A, B, or C), coagulopathy, fish consumption >2 times/week, treatment with steroids, allergy to fish.
**Eritsland**^[55]^, RCT – SB, **OP**: CABG | **CPB**: 100%, PUFA: 260, Ctrl: 251, **Sum**: 511, **Mean age**: 60 ± 9, *♂*: 347/610 (56.9%)	**IC**: scheduled CABG
	**EC**: Death – angiography before 9 months. Antidiabetic/lipid lowering therapy during the study period. Failure to attend 9 months visit. Deviation from the assigned treatment,

There are only a handful of studies (14 out of 31 included) that give us an insight about the changes in plasma or atrial n-3 PUFAs concentrations after substitution followed by adult cardiac or more specifically, in the majority, after CABG surgery (Table 4). In the majority of these studies, the levels of plasma n-3 PUFA were measured preoperatively as baseline measurements, and followed up for up to 4 days and in one case 21 days^[44]^ postoperatively. There were only two studies that looked at the n-3 PUFA concentrations for 6^[45]^ and 9^[46]^ months following heart surgery. The pattern of plasma n-3 PUFA concentration changes postoperatively in most of the studies is surprisingly the same. In about half of these studies, there is a 10–50% postoperative decrease in the n-3 PUFA levels in the control group which is attributed to the consumption of plasma/atrial n-3 PUFA antioxidants^[44,47-49]^ after CPB (Cardio Pulmonary Bypass). In the rest of the studies examined, CPB does not seem to affect the postoperative plasma/tissue n-3 PUFA levels in the control groups.^[45,48,50-56]^ Plasma n-3 PUFA levels correlated strongly with right atrial tissue n-3 PUFA levels^[54]^. Compared to placebo or controls there is an average increase up to 40–50% in plasma, platelet, erythrocyte and atrial tissue n-3 PUFA levels in the intervention group,^[44,46,50-53,56,57]^ often more accentuated in EPA percentage/concentration^[45,46,50,56]^. In contrast to the majority of authors, Heidarsdottir *et al*^[47]^ observed a peak n-3 PUFA measurement only perioperatively, while the control group had lower n-3 PUFA levels, both on the day of the operation (POD 0) and POD3. Akintoye *et al*^[49]^, examined monoepoxides derived from polyunsaturated fatty acids (MEFAs) and their response to n-3 PUFA supplementation and found that compared with placebo at POD2, levels of EPA- and DHA-derived MEFAs were 40% and 18% higher, respectively, and that the n-3 PUFA supplementation significantly ameliorated the reduction in postop n-3 MEFAs, while the degree of increase in n-3 MEFAs related positively to the circulating level of their n-3 PUFA precursors^[49]^. Levels of atrial n-3 PUFA were measured in just a few of the aforementioned studies and this was only done in a single timepoint, namely atrium sampling during surgery^[48,50,54,56]^. In consistent with plasma levels findings, intervention group resulted in significant increase of n-3 PUFA in atrial tissue for up to 40–50%, a finding which was more accentuated in the EPA group^[56]^. For more clarity, we conducted a graphical representation with clustered boxplots (Figs. 2-7) of the studies’ values presented in Table 4, after allocating them to comparable groups [Group 1^[44,48,50-53,56,57]^: Intervention vs control group measurement of n-3 PUFA percentage of FA in plasma or atrium or red blood cells – RBC in different timepoints T1-T2-T3; Group 2^[45,47,55]^: Intervention vs control group measurement of n-3 PUFA in mg/L in plasma or atrium or RBC in different timepoints T1-T2-T3; Group 3^[54,58]^: Postoperative atrial fibrillation group (POAF) and non-POAF (NOPOAF) group measurement of n-3 PUFA percentage of FA in plasma or atrium or RBC in different timepoints T1-T2-T3]. Interestingly, mean comparison^[41]^ and subsequent ANOVA^[42]^ between various groups and timepoints revealed a statistically significant difference in mean percentage of n-3 PUFA between different time points [T0-T1*-T2] (*P* = 0.003) in group 1. In contrary, no statistically significant difference in n-3 PUFA level was proven, neither in group 2 (*P* = 0.29) nor group 3 (*P* = 0.64). Unlike the rest authors, Gu *et al*^[54]^ did not find any differences, neither in the atrial groups, nor in the plasma groups, though in this study measurements were taken only during surgery and there are no timewise comparisons. Noteworthy is that they found significant correlations between the content of total n-3 PUFA, EPA and DHA (but not DPA), in atrial tissue and plasma phospholipids. We hereby assume that since essential n-3 PUFA are not synthetized in humans, their low plasma or atrial levels postoperatively,^[44,47-50,56,58]^ will persist for weeks or even for longer periods if patients receive inadequate amounts of n-3 PUFA through their diet or through supplementation. This can inevitably predispose this group of patients to the risks accompanying n-3 PUFA low plasma/tissue levels or even deficiency. Based on current bibliographic evidence, in Table 2, we summarize the consequences of n-3 PUFA deficiency on different systems and organs.

**Table 4:** Studies assessing changes in n-3 PUFA levels following adult CS.


Study N-3 PUFA regimen in intervention vs control group	N-3 PUFA measurement in plasma or atrium in mean % of fatty acids (FA), or μg or mol ± standard deviation (SD) or range	Timepoints (T0-T1-T2) of N-3 PUFA sample measurement			*P*-value (T0 vs T1 vs T2)
		T0 – Baseline	T1 – Surgery	T2 – POD1/4	
**Gu**^[54]^ POAF: 18 NOPOAF: 31	Plasma %N3FA/FA (SD) [POAF]	–	6.38 (1.47)	–	–
	Plasma Ctrl %N3FA/FA (SD) [non POAF]	–	6.36 (1.78)	–	–
	Atrial %N3FA/FA (SD) [POAF]	–	3.89 (0.65)	–	–
	Atrial Ctrl %N3FA/FA (SD) [non POAF]	–	3.91 (1.08)	–	–
**Saravanan**^[50]^ I: 52 C: 51 PO, 15 d, 2 g/d, (5 d preop – discharge).	Plasma %EPA/FA (SD)	1.51 (0.98)	2.78 (1.13)^a^	**–**	SS
	Plasma Ctrl %EPA/FA (SD)	1.76 (1.49)	1.53 (0.74)	**–**	NS
	Plasma %DHA/FA (SD)	3.79 (1.37)	5.49 (1.02)^a^	**–**	SS
	Plasma Ctrl %DHA/FA (SD)	3.82 (1.38)	4.19 (1.29)	**–**	NS
	Plasma %N3FA/FA (SD)	5.3 (1.68)	8.27 (1.52)^a^	–	SS
	Plasma Ctrl %N3FA/FA (SD)	5.58 (2.03)	5.72 (1.49)	–	NS
	Atrial %N3FA/FA (SD)	–	17 (4.02)^a^	**–**	**–**
	Atrial Ctrl %N3FA/FA (SD)	–	13.3 (3.8)	–	**–**
**Rubanenko**^[44]^ I: 148 C: 158 PO, 26 d, 2 g/d 5 d preop & 1 g/d 21 d postop	Plasma %EPA/FA (range)	0.74 (0.19–1.82)	–	1.11 (0.3–1.72) [21POD]	NS – 0.29
	Plasma Ctrl %EPA/FA (range)	0.64 (0.41–1.05)	–	0.48 (0.2–0.9) [21POD]	NS – 0.14
	Plasma %DHA/FA (range)	4.23 (1.2–5.86)	–	6.2 (3.83–7.95)^a^ [21POD]	SS – 0.01
	Plasma Ctrl %DHA/FA (range)	5.36 (2.55–6.22)	–	2.79 (0.78–5.55) [21POD]	NS – 0.08
	Plasma %N3FA/FA index (range)	3.84 (1.23–6.33)	–	6.89 (4.82–9.64)^a^ [21POD]	SS – 0.01
	Plasma Ctrl %N3FA index (range)	5.29 (2.59–6.85)	–	3.9 (1.02–6.9) [21POD]	SS – 0.02
**Heidarsdottir**^[47]^ I: 83 C: 85 PO, 17 d, 224 g/d, (5-7 d preop – discharge).	Plasma N3FA μg/mL (range)	102.5 (49.3–237.5)	115.4 (70.9–256.1)^a^	101.4 (45.8–187.4)^a^	SS: (0/1. 1/2)
	Plasma Ctrl N3FA μg/mL (range)	102.3 (51.0–224.7)	88.5 (47.3–193.7)	89.8 (43.5–187)	SS: (0/1. 0/2)
**Skuladottir**^[58]^ I: 62 C: 63 PO, 1 w preop, 2.24 g/d,	Plasma %EPA/FA (SD) [POAF]	3.45 (0.22)	–	2.77 (0.17)^a^	SS
	Plasma Ctrl %EPA/FA (SD) [non POAF]	3.25 (0.19)	–	2.33 (0.14)	SS
	Plasma %DHA/FA (SD) [POAF]	6.88 (0.16)^a^	–	6.93 (0.15)^a^	NS
	Plasma Ctrl %DHA/FA (SD) [non POAF]	6.35 (0.15)	–	6.40 (0.14)	NS
	Plasma %N3FA/FA (SD) [POAF]	11.71 (0.14)^a^	–	10.8 (0.26)^a^	SS
	Plasma Ctrl %N3FA/FA (SD) [non POAF]	10.83 (0.28)	–	9.75 (0.17)	SS
**Andreassen**^[45]^ I: 14 C: 14 PO, 6 m, 4 g/d (d4–6 m)	Plasma N3FA (mg/L) (SD)	104 (9.22)	–	230 (12.2)^a^ [6 moPO]	SS
	Plasma Ctrl N3FA (mg/L) (SD)	143 (11.66)^a^	–	164 (12.80) [6 moPO]	NS
**Sandesara**^[51]^ I: 120 C:123 PO, 16 d, 4 g/d (2 d preop – 14 d postop)	Plasma %N3FA/FA (range)	2.89 (2.66–3.11)	4.35 (4.00–4.70)^a^	4.27 (4.02–4.51)^a^	SS: (0/1. 0/2)
	Plasma Ctrl %N3FA/FA (range)	2.88 (2.68–3.09)	2.78 (2.61–2.95)	3.03 (2.85–3.21)	NS
**Eritsland**^[55]^ I: 256 C: 251 PO, 9 m, 4 g/d, 2d post to 9 m post.	Plasma N3FA (mg/L) (SD)	177.5 (55.9)	–	256.9 (57.7) [9 moPO]	SS
	Plasma Ctrl N3FA (mg/L) (SD)	170.3 (51.4)	–	168.5 (56.8) [9 moPO]	NS
	Plasma EPA (mg/L) (SD)	38.9 (24.1)	–	93.2 (29.8) [9 moPO]	SS
	Plasma Ctrl EPA (mg/L) (SD)	33.5 (19.9)	–	34.5 (23.5) [9 moPO]	NS
	Plasma DHA (mg/L) (SD)	113.1 (30.9)	–	129.1 (26.4) [9 moPO]	SS
	Plasma Ctrl DHA (mg/L) (SD)	111.4 (30.8)	–	107.0 (32.0) [9 moPO]	NS
**Farquharson**^[48]^ I: 97 C: 97 PO, 1 m, 4.6 g/d, (3 w preop – discharge)	RBC %N3FA/FA (SD)	8.73 (1.14)	12.0 (1.9)^a^	–	SS
	RBC Ctrl %N3FA/FA (SD)	8.75 (0.95)	8.58 (0.98)	–	NS
	Atrial %N3FA/FA (SD)	–	9.99 (1.78)^a^	–	–
	Atrial Ctrl %N3FA/FA (SD)	–	6.59 (1.31)	–	–
**Akintoye**^[49]^ (2016) I: 249 C: 230 PO, 15 d, 10 g/d (2–5 d pre) & 2 g/d (10 d post)	Plasma EPA MEFA (nmol/L) (SD)	27 (13)	–	7.0 (–)^a^	SS
	Plasma Ctrl EPA MEFA (nmol/L) (SD)	26 (13)	–	5.0 (2.5)	SS
	Plasma DHA MEFA (nmol/L) (SD)	212 (164)	–	104 (–)^a^	SS
	Plasma Ctrl DHA MEFA (nmol/L) (SD)	270 (204)	–	88 (51)	SS
**Akintoye**^[57]^ (2018) I: 758 C: 758 PO, 15 d, 10 g/d (5 d pre), 2 g/d (10 d post)	Plasma %N3FA/FA (SD)	4.66 (0.12)	5.62 (0.12)	–	SS
	Plasma Ctrl %N3FA/FA (SD)	–	–	–	–
**Jackson**^[52]^ I: 159 C: 161 PO, 15 d, 10 g/d (2–5 d pre) & 2 g/d (10 d post)	Plasma %N3FA/FA (SD)	4.226 (1.01)	5.86 (1.07)^a^	–	SS
	Plasma Ctrl %N3FA/FA (SD)	4.187 (1.14)	4.252 (1.08)	–	NS
**Mozaffarian**^[53]^ I: 758 C: 758 PO, 15 d, 10 g/d (2–5 d pre) & 2 g/d (10 d post)	Plasma %N3FA/FA (SD)	4.65 (1.44)	6.40 (1.70)^a^	–	SS
	Plasma Ctrl %N3FA/FA (SD)	4.65 (1.43)	4.78 (1.43)	–	NS
**Berger**^[56]^ I: 14 C: 14 IV, 14 hrs, 0.2 g/kg (12 hrs pre & 2 hrs post)	Platelets %EPA/FA (mol) (SD)	0.4 (0.2)	–	1.3 (0.3)^a^	SS
	Platelets Ctrl %EPA/FA (mol) (SD)	0.12 (0.1)	–	0.2 (0.2)	NS
	Platelets %DHA/FA (mol) (SD)	2.14 (0.4)	–	3.2 (0.7)^a^	SS
	Platelets Ctrl %DHA/FA (mol) (SD)	2.25 (0.5)	–	2.4 (1.1)	NS
	Atrial %EPA/FA (mol) (SD)	–	1.0 (0.2)^a^	–	–
	Atrial Ctrl %EPA/FA (mol) (SD)	–	0.5 (0.2)	–	–
	Atrial %DHA/FA (mol) (SD)	–	6.8 (1.0)	–	–
	Atrial Ctrl %DHA/FA (mol) (SD)	–	6.7 (1.3)	–	–

hrs: hours, d: days, m: months, g/gr: milligrams, kg: kilogram: PO: orally, IV: intravenously, preop/pre: preoperatively, postop/post: postoperatively, SD: standard deviation, L/L: liters, mol: moles, POAF: postoperative atrial fibrillation, 6moPO: 6 months postoperatively, I: intervention, C/Ctrl: control, FA: fatty acids, MEFA: monoepoxides from FA

Difference between timepoints (T0-T1-T2): Statistically significant (SS), non-Statistically significant (NS), (-): non defined

^a^ Significant difference between timepoint measurement vs respective control (Ctrl).

**Figure 2.**
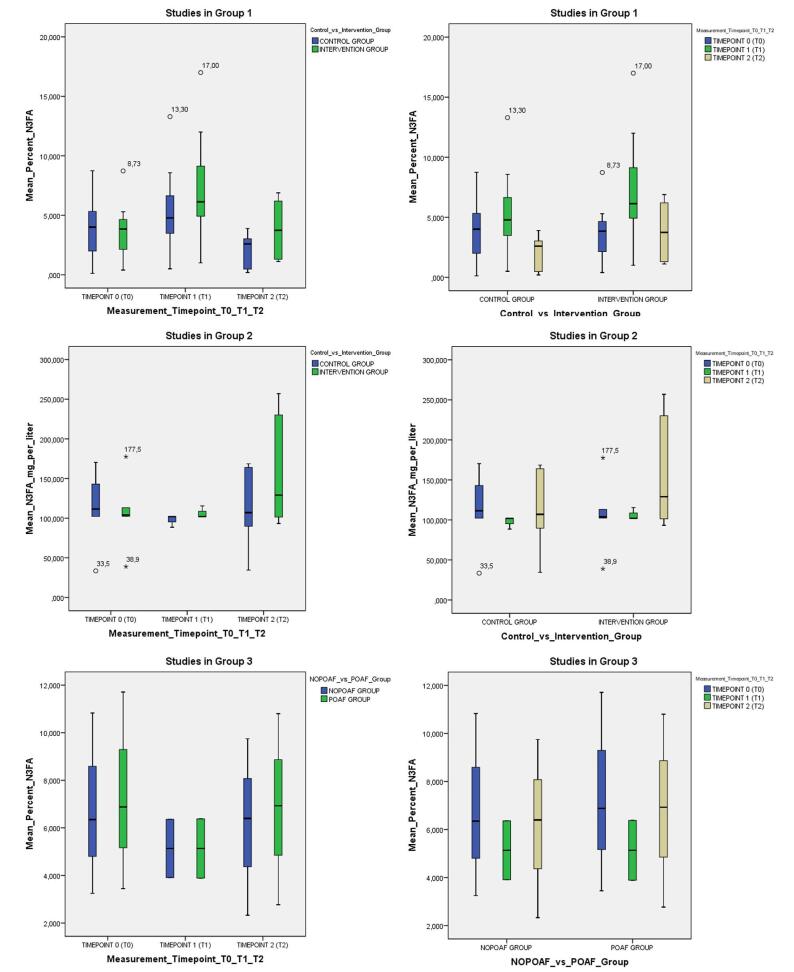
Graphical representation with clustered box plots of the studies' values presented in Table 4, after allocating them in comparable groups.**Group 1**^[44,48,50-53,56,57]^: Intervention vs control group measurement of n-3 PUFA percentage of FA in plasma or atrium or red blood cells - RBC in different timepoints T1-T2-T3; **Group 2**^[45,47,55]^: Intervention vs control group measurement of n-3 PUFA in mg/L in plasma or atrium or RBC in different timepoints T1-T2-T3; **Group 3**^[54,58]^: Postoperative atrial fibrillation group (POAF) and non-POAF (NOPOAF) group measurement of n-3 PUFA percentage of FA in plasma or atrium or RBC in different timepoints T1-T2-T3.

### Characteristics and clinical settings of n-3 PUFA studies

N-3 PUFA antioxidant and other properties (Table 1) in the context of CS, has been under investigation since the late 90s. In our review, the literature extends from 1995 up to 2022. The geographic expansion of the studies includes almost all continents (North America, South America, Europe, Asia, and Australia). Notably there are far many studies from Europe and specifically from Italy. To our knowledge, there is no other review on the subject that expands within a 27-year timeframe. All elective adult CS were included, but the vast majority of articles identified were studies performed on patients that had CABG operation with CPB (Table 3-5).

**Table 5:** Characteristics of studies included in the review.

Author, year, country	Results
Study regimen (StR)	
Outcome measures (OM)	Comments
**Calo**^[65]^, 2005, Italy	POAF: PUFA: 12/81–15.2% | Control: 27/81–33.3%, OR 0.35; 95% CI 0.16–0.76, *P* = 0.013 HLOS (d): PUFA: 7.3 ± 2.1 | Control: 8.2 ± 2.6, *P* = 0.017, Postop Complications: PUFA: 6.3% | Control: 8.6%, *P* = 0.8. Postop Mortality: PUFA: 1.3% | Control: 2.5%, *P* = 1.0. Predictors of POAF: 2 variables were noted to be significant independent predictors of POAF: age (OR 1.08; 95% CI 1.01 to 1.15; *P* = 0.022) & the use of PUFAs (OR 0.32; 95% CI 0.10 to 0.98; *P* = 0.013). The use of PUFAs during hospitalization in patients undergoing CABG significantly reduced the incidence of POAF (18.1% absolute risk reduction, 54.4% relative risk reduction) and was associated with a shorter HLOS. Except for a single case of allergic response, no significant adverse reactions were observed.
**StR**: n-3 PUFAs 2 g/day PO 5 days preop until discharge from the hospital. The administration of PUFAs in the immediate postop period (24–36 h) was done if needed through a nasogastric tube. B-blockers: PUFA: 56.8% | Control: 34.8%	
**OM**: POAF, HLOS, postop complications	
**Saravanan**^[50]^, 2009, UK	POAF: PUFA: 56% | Control: 43%, *P* = 0.28, 95% CI −6 to 30%, Clinical AF: PUFA: 42% | Control: 35%, *P* = 0.60, AF Burden (h): PUFA: 13.2 | Control: 9.6, *P* = 0.49, HLOS (d): PUFA: 8.5| Control: 7, *P* = 0.49, ICULOS: NS differences, CRP: NS differences at either time point (24 h & POD3). Postop complications: NS differences. Median dietary n-3 PUFA Intake (baseline): PUFA: 131 mg | Control: 118 mg. In conclusion, n-3 PUFA given at a dose of 2 g/d increased the n-3 PUFA serum and atrial tissue content but did not reduce the incidence of AF in the 5 days after CABG. In addition, there appeared to be a trend toward more AF in the n-3 PUFA group. N-3 PUFA Content of Serum and Right Atrial Appendage Tissue: Serum PC EPA and DHA did not differ between the 2 groups at study entry and did not change in the placebo group. However, both EPA and DHA in serum PC increased significantly in the n-3 PUFA group and were higher at surgery in the n-3 PUFA group than in the placebo group. Importantly, PC and PE EPA and DHA in atrial tissue collected at surgery were higher in the n-3 PUFA group than in the placebo group.
**StR**: PUFA 2 g/d PO or olive oil for at least 5 days preop and until hospital discharge.	
**OM**: POAF, HLOS, ICULOS, CRP, n-3 PUFA Content of Serum and Right Atrial Appendage Tissue (fatty acid compositions of serum phosphatidyl-choline (PC), the major circulating phospholipid, and of atrial tissue PC and phosphatidyl-ethanolamine (PE), Dietary n-3 PUFA Intake, Postop Complications	
**Heidt**^[11]^, 2009, Germany	POAF: PUFA: 9/52–17.3% | Control: 15/50–30.6%, *P* < 0.05, ICULOS & HLOS: shorter in PUFA group. Patients who converted to AF had a trend to remain longer in the ICU and to have a longer HLOS compared to patients in SR. This study showed that oral PUFA reduce the length of stay in the hospital to a range similar to that of patients without atrial fibrillation. Intravenous administration is advisable, to prevent the problem of fluctuating bioavailability. Due to the proven beneficial effect on perioperative atrial fibrillation, PUFA are recommended for the perioperative therapy of patients undergoing CABG. Preop IV infusion of PUFA should be recommended.
**StR**: 100 mg soya oil/kg body weight/day IV (control group) or 100 mg fish oil/kg body weight/day IV (intervention) starting 12 h preop until discharge from ICU.	
**OM**: POAF, ICULOS, HLOS	
**Heidarsdottir**^[47]^, 2009, Iceland	POAF: PUFA: 54.2% | Control: 54.1%, *P* = 0.99. Factors associated with POAF: there was no association between POAF and assignment to PUFA treatment, plasma levels of n-3 PUFA, use of b-blockers, or other variables tested. However, advanced age, peak postop CRP level, lower BMI, an operative procedure other than a simple CABG, and non-smoking were associated with a higher risk of developing POAF. Effect of PUFA in POAF: no differences in the incidence of POAF were found between groups when the study cohort was divided into tertiles of n-3 PUFA blood levels at baseline and at the day of surgery, and of peak postop CRP levels. The concentration of total plasma n-3 PUFA on the day of surgery was higher in patients with POAF than those without POAF (*P* = 0.006). Median time to the development of POAF (h): PUFA: 49 | Control: 45, *P* = 0.42, Postop outcomes (blood loss, red blood cell transfusions, frequency of re-operation, major bleeding): NS differences. Concentration of n-3 PUFA in plasma phospholipids (baseline, operation day & POD3): The total concentration of n-3 PUFA in plasma phospholipids in PUFA group increased from a baseline of 102.5 to 115.4 mg/mL on the day of surgery (*P* = 0.002), whereas in the placebo group it decreased from 102.3 to 88.5 mg/mL (*P <* 0.001). At operation day & POD3 PUFA levels were higher in PUFA group compared to placebo group (*P* ≤ 0.001). AE: self-reported gastrointestinal discomfort: PUFA: 13.2% | Control: 3.5%, *P* = 0.02. Overall, PUFA treatment was well tolerated without serious AEs compared with placebo. There is no evidence for a beneficial effect of treatment with n-3 PUFA on the occurrence of POAF in patients undergoing open CS.
**StR**: 1240 mg EPA & 1000 mg DHA or 2 g olive oil (placebo) PO for 5–7 days preop & postop until hospital discharge or for a max duration of 2 weeks following surgery in patients with prolonged hospital stay.	
**OM**: POAF, postop outcomes (blood loss, red blood cell transfusions, frequency of re-operation)	
**Sandesara**^[51]^, 2012, USA	POAF: PUFA: 36/120–30% | Control: 40/123–33%, *P* = 0.67, OR = 0.89, 95% CI 0.52–1.53, Mean duration of POAF (h): PUFA: 7.9 | Control: 10.6, *P* = 0.68, HLOS: NS differences between groups (*P* = 0.27). Postop Complications (including death): NS differences. Plasma Fatty Acids: At the time of screening: no differences. At day of surgery & POD 4, mean plasma EPA + DHA levels were significantly higher in the n3-PUFA group (both *P <* 0.001) and the n6:n3-PUFA ratio in n3-PUFA group was lower than in the placebo group (both *P <* 0.001). Mean n6:n3-PUFA ratio: POAF: 7.7 (95% CI 6.9–8.6) | No POAF: 7.6, *P* = 0.76, 95% CI 7.1–8.0. Mean Heart Rate (bpm): PUFA: 84.5 ± 11.0 | Control: 85.0 ± 10.9, *P* = 0.72. There were NS differences in plasma fatty acid levels in patients who had POAF compared to those who did not. The present study documented increased plasma PUFA levels with n3-PUFA administration, but the increase in fatty acid levels did not reduce the incidence of POAF. Oral n3-PUFA supplementation did not reduce AF or other complications postop.
**StR**: n3-PUFA 2 g PO twice daily (minimum of 6 g) ≥ 24 hours preop until the primary endpoint (clinically significant AF requiring treatment) or for a maximum of 2 weeks postop.	
**OM**: POAF, 30 d mortality, plasma n6:n3-PUFA ratio & plasma EPA + DHA levels.	
**Farquharson**^[48]^, 2011, Australia	POAF: PUFA: 36/97–37% | Control: 47/97–48%, *P* = 0.11, Time to first occurrence of AF: delayed time to AF in PUFA group compared to the control group, *P* = 0.06, HR 0.66 95% CI 0.43–1.01, ICULOS (h): PUFA: 67 ± 52 | Control: 95 ± 158, CI 0.56–0.90, *P* = 0.005, HLOS (d): PUFA: 8.6 ± 7.1 | Control: 9.9 ± 10.2, *P* = 0.12, AE: NS diff. Rate of major bleeding: lower in PUFA group, *P* = 0.21 Rate of red blood cell transfusion: PUFA: 25/97–26% | Control: 42/97–43%, *P* = 0.02, Red blood cell phospholipid fatty acids: at the time of surgery, PUFA group had significant increases from baseline in red blood cell EPA & DHA and a significant decrease in n-6 PUFAs, whereas there were NS changes in the control group. Atrial phospholipid fatty acids: PUFA group had significantly larger proportions of EPA & DHA and a significantly lower proportion of arachidonic acid in atrial tissue than the control group. Perioperative treatment with high-dose fish oil failed to significantly decrease the incidence of POAF (NS trend for reduction), but there was a significant decrease in ICULOS and a NS trend for shorter HLOS.
**StR**: 15 mL/day oil PO (4.6 n-3 PUFAS: EPA acid 2.7 g/day & DHA acid 1.9 g/day) or oil (control) starting 3 weeks preop until POD6 or until discharge (whichever came first).	
**OM**: POAF (for 6 POD), ICULOS, HLOS, AE	
**Veljović**^[82]^, 2013, Serbia	Blood loss (POD1): PUFAs: 680 ± 274 mL | Control: 608 ± 210 mL, *P* = 0.356, NS. Platelet aggregation: NS different between PUFAs and control group expect COL test. Adenosine diphosphate (ADP) test: PUFA: 39 ± 11 | Control: 42 ± 15, *P* = 0.701. Arachidonic acid (ASPI) test: PUFAs: 64 ± 24 | Control: 70 ± 27, *P* = 0.525. Thrombin receptor-activating peptide (TRAP) test: PUFAs: 68 ± 25 | Control: 75 ± 26, *P* = 0.396. Collagen (COL) test aggregation: PUFAs: 32 ± 15 | Control: 47 ± 20, *P* = 0.009. Transfusion requirements and postop blood loss: NS diff. Postop complications: similar in both groups. Acute pretreatment with PUFAs did not affect INR, aPTT and postop bleeding volume. The activity of platelets was statistically significant lower postop in both groups, particularly markedly pronounced in the COL test in the PUFAs group, but with no negative effect on bleeding.
**StR**: IV infusion of 100 mL n-3 PUFAs or 0.9% saline solution (control group) 1 day preop & 4 hours before CPB.	
**OM**: hematological parameters and platelets aggregation using platelet aggregation tests: arachidonic acid (ASPI test), adenosine diphosphate (ADP test), thrombin receptor-activating peptide (TRAP test) and collagen (COL test).	
**Sorice**^[66]^, 2011, Italy	POAF: G1: 11.1% | G2: 11.7% | G3: 12.5% | G4: 31.6%. POAF: PUFA (G1&2): 11/96–11.4% | Controls (G3&4): 24/105–22.8%, OR 0.43; 95% CI 0.2–0.95; *P* = 0.033, HLOS: NS differences (*P* = 0.75). Postop complications: off-pump: 10.7% | on-pump: 11.1%, *P* = ND. PUFA administration significantly reduces the overall incidence of POAF in patients undergoing CABG (11.4% absolute risk reduction, 49.8% relative risk reduction). A statistically significant reduction of POAF was observed only in patients undergoing on pump CABG (19.8% absolute risk reduction, 62.7% relative risk reduction). Scientists are increasingly supporting the hypothesis of an important role of surgery-related inflammatory cascade as the leading cause of post-operative AF. Present findings may further support the hypothesis of an anti-inflammatory PUFA action considering that “on-pump” CABG induces a systemic inflammatory response by triggering the production and release of inflammatory mediators.
**StR**: n-3 PUFAs 2 g/day PO for at least 5 days preop until hospital discharge. Group 1 (G1 =: PUFA off-pump (n = 45). Group 2 (G2): PUFA on-pump (n = 51). Group 3 (G3): no PUFA off-pump (n = 48). Group 4 (G4): no PUFA on-pump (n = 57)	
**OM**: POAF, impact of the surgical technique (off-pump/on-pump) on the incidence of POAF, HLOS.	
**Farahani**^[67]^, 2017, Iran	POAF: PUFA: 17/201–8.40% | Control: 29/199–14.07%, *P* = 0.07, OR 0.56, Mean episodes of AF: PUFA:2 | Control: 3.5, *P* = 0.06, OR −1.75. Mean total duration of AF (h): PUFA: 20.96 ± 4.71 | Control: 46.87 ± 7.44, *P* = 0.04. Time to AF improvement: shorter in intervention group, HR: 2.05; 95% CI = 0.70–6.22, *P* = 0.20. ICULOS (h): PUFA: 63.1 ± 2.0| Controls: 74.3 ± 4.1, *P* = 0.003. HLOS (mean d): PUFA: 14| Controls: 14, *P* = 0.04, OR −0.83. PUFA consumption leads to a shorter time to AF improvement (NS). The incidence of AF in patients undergoing CABG while taking PUFA was decreased by approximately 5.7% (SS). Fish oil reduced HLOS & ICULOS.
**StR**: 2 g/d fish oil (300 mg EPA & 200 mg DHA) PO or placebo PO (olive oil) for at least 5 days preop until hospital discharged.	
**OM**: POAF, time of AF improvement, need of pharmacologic therapy or cardioversion, HLOS, ICULOS	
**Gholami**^[78]^, 2019, Iran	Fatigue score: Intervention: 62.01 ± 4.06 | Control: 67.92 ± 4.95, *P <* 0.000, Changes in general fatigue score: Intervention: 1.42 ± 1.15 | Control: 2.66 ± 2.50, *P <* 0.000, Changes in physical fatigue score: Intervention: 1.99 ± 0.23 |Control: 2.26 ± 1.53, *P <* 0.000, Changes in reduced active score: Intervention: 1.83 ± 0.14| Control: 1.83 ± 0.14, *P* = 0.0, Changes in mental fatigue score: Intervention: 3.00 ± 2.16| Control: 3.32 ± 2.44, *P* = 0.586. Administration of n-3 PUFA associated with vitamin C as supplements can effectively reduce general and physical fatigue as well as the reduced activities and motivation to live among CABG patients.
**StR: Preop**: PUFA 2 g/bid & Vit C 1 g/bid the day before op. **Post**: 2 g PUFA (1 g/bid) & 1 g VitC (500 mg/bid) until 5 POD. **Route**: via nasogastric tube and then orally.	
**OM**: Fatigue score – MFI-20 scale	
**Rodrigo**^[61]^, 2013, USA	POAF: Intervention: 10/13–9.7%| Control: 32/100–32%, RR 0.28; 95% CI: 0.14–0.56; *P* < 0.001. Mean duration of POAF (h): Intervention: 10.8 ± 0.7| Control: 11.2 ± 1.5, *P* = 0.06. The placebo patients had 3.62 times more risk for POAF at any day compared with the supplemented patients (HR 3.62; 95% CI: 1.78 to 7.36; *P* < 0.001). Time of POAF occurrence (d): Intervention: 3.3 ± 0.7 | Control: 2.9 ± 0.4, Spontaneous cardioversion in SR: Intervention: 3| Control: 4, *P* = 0.20. ICULOS (d): Intervention: 2.87 ± 0.44| Control: 3.08 ± 0.54, *P* = 0.76. AE: Intervention: 7/103 |Control: 7/100, *P* = 0.586. HLOS (d): Intervention: 8.77 ± 0.37|Control: 9.57 ± 0.66, *P <* 0.05. Oxidative stress & Inflammation: postop placebo patients presented with increased levels of biomarkers of inflammation and oxidative stress, which were markedly attenuated by antioxidant supplementation. The activity of catalase, superoxide dismutase, and glutathione peroxidase in atrial tissue of the supplemented patients was 24.0%, 17.1%, and 19.7% higher than the respective placebo values (*P* < 0.05). The atrial tissue of patients who developed AF showed NADPH oxidase p47-phox subunit protein and mRNA expression 38.4% and 35.7% higher, respectively, than patients in SR (*P* < 0.05). This safe, well-tolerated, and low-cost regimen favorably affected POAF, increased antioxidant potential, and attenuated oxidative stress and inflammation.
**StR**: N-3 PUFA 2 g/day 7-day preop & 2 days preop added VitC 1 g/day and VitE 400 IU/day until discharge.	
**OM**: POAF, biomarkers of oxidative stress (MDA levels) and inflammation (CRP, leucocytes).	
**Veljović**^[80]^, 2013, Serbia	Lactate extraction: 10 & 20 min after aortic cross-clamp time has shown negative values in the control group, but positive values in the PUFAs group with statistically significant differences (−19.6% vs 7.9%, *P* < 0.0001 and −19.9% vs 8.2%, *P* < 0.0008, respectively). Lactate extraction 30 min after reperfusion was not statistically different between the two groups (6.9% vs 4.2%; *P* < 0.54). Oxygen extraction: in PUFA group was significantly higher compared to control group after 10, 20 & 30 min of reperfusion (35.5% vs 50.4%, *P* < 0.0004; 25.8 % vs 48.7%, *P* < 0.0001 and 25.8% vs 45.6%, *P* < 0.0002, respectively). TnT level: 4 & 24 h after CPB was significantly lower in PUFA vs ctrl group being 72% (11.4 ng/mL: 6.67 ng/mL, *P <* 0.009) and 115% (12.7 ng/mL: 5.9 ng/mL, *P <* 0.008) lower at each time point respectively. CK-MB level: 4 h after CPB: significantly lower in PUFA group (61.9 vs 37.7, *P* < 0.008) | 24 h after CPB: NS differences between two groups (58.9 vs 40.6, *P* < 0.051). Postop complications: NS differences. Extraction of oxygen and the uptake of lactate were markedly increased in the PUFA pretreated patients compared to the control group, with the subsequent decrease of serum TnT and CK-MB levels in the PUFAs group, pointing thus to their important cardioprotective effect. Acute IV administration of PUFA was associated with a significant reduction in myocardial ischemic-reperfusion injury. Preop treatment with n-3 PUFAs promoted early metabolic recovery of the heart after elective CABG and improved myocardial protection.
**StR**: Preop (1 d & 4 h before CPB) PUFA IV infusion 100 mL (25 mL/h) or 0.9% saline solution.	
**OM**: lactate extraction /excretion, myocardial oxygen extraction, troponin I (TnT), creatine kinase–myocardial band (CK-MB).	
**Akintoye**^[57]^, 2018, USA, Italy, Argentina	BARC type 4 or 5 bleeding: PUFA: 5.51% | Control: 6.61%, *P* = 0.34, OR = 0.81, CI = 0.53–1.24. TIMI major bleeding: PUFA: 4.12% | Control: 5.51%, *P* = 0.16; OR = 71, CI = 0.44–1.14. Total units of blood transfused: PUFA: 1.6 | Control: 1.92, *P <* 0.001. Mean 24-h chest tube output (mL): PUFA: 388 | Control: 370, *P* = 0.48. Association Between Circulating Plasma PUFA Levels and Risk of BARC Type 4 or 5: achieved levels of PUFA on the morning of CS associated significantly with lower risk of bleeding, with 70% lower risk in the third quartile (OR, 0.30, 95% CI, 0.11–0.78) and 64% lower risk in the fourth quartile (0.36, 95% CI, 0.15–0.87), compared with the lowest quartile (*P* = 0.01). Independent predictors of BARC type 4 or 5 bleeding: cardiopulmonary bypass use – the strongest predictor (OR, 5.65; 95% CI, 1.72–18), followed by valvular surgery (±concomitant CABG), preop antithrombotic therapy, female sex, and presence of diabetes mellitus. Fish oil supplementation did not increase perioperative bleeding and reduced the number of blood transfusions. Higher achieved n-3-PUFA levels were associated with lower risk of bleeding. These novel findings support the need to reconsider current recommendations to stop fish oil or delay procedures before CS.
**StR**: 8–10 g PUFA PO (1 g = 465 mg of EPA + 375 mg) for 2–5 days preop & 2 g/d postop until discharge or POD 10 (whichever came first).	
**OM**: major perioperative bleeding (BARC type 4 or 5), perioperative bleeding per thrombolysis in myocardial infarction (TIMI), chest tube output, total units of blood transfused	
**Lomivorotov**^[91]^, 2014, Russia	POAF 10 d follow-up: PUFA: 27.8% | Control: 19%, *P* = 0.88. POAF 6/12/24 months follow-up: PUFA: 35.3% | Control: 27.8% There was NS difference between the 2 curves (*P* = 0.64); however, there was trend toward higher probability of AF in PUFA group. POAF 2-year follow-up: PUFA: 35.3% | Control: 27.8%, *P* = 0.9. AF duration predicted risk of cardiovascular hospitalization at the 2-year follow-up (regression coefficient estimate = 0.24, standard error 0.02, *P* < 0.0001; *R*^2^ = 0.74). Intergroup differences in secondary end points were not found. Groups were comparable by postop clinical characteristics (ventilation time, inotropic support, ICULOS, HLOS). AE: no event of bleeding or infection was detected. New-onset AF during 10-day postop significantly predicted readmissions (OR 78; 95% CI 6–1008; *P* = 0.0008). Infusion of PUFA failed to prevent the occurrence of AF 2 years after CABG. The cumulative AF duration registered by the continuous cardiac monitor at the 2-year follow-up was a significant predictor of an adverse outcome. Early new onset POAF was associated with significant morbidity (cerebrovascular events, readmission to the hospital due to AF, and heart failure) 2 years postop due to the high risk of reoccurrence of AF.
**StR**: IV n-3 PUFA 200 mg/kg/day starting 24 h before anesthesia induction followed by 100 mg/kg/day from POD2 to POD10. Cardiac monitor was implanted sc.	
**OM**: POAF or other atrial arrhythmias (2-year follow-up). Secondary end points: cardiac troponin I, C-reactive protein, interleukin6, interleukin10, brain natriuretic peptide (BNP), proatrium natriuretic peptide (pro-ANP), adverse cardiovascular events.	
**Skuladottir**^[58]^, 2011, Iceland	POAF: 62/125–49.6%. POAF increased significantly with each higher quartile of pre- and postoperative docosahexaenoic acid (DHA) and diminished significantly with each higher quartile of pre- and postoperative arachidonic acid (AA). POAF group was older (*P* = 0.003) and had lower BMI (*P* = 0.026). Median CRP levels (mg/L): PUFA: 218 | Controls: 201, *P <* 0.05. Postop characteristics (ICULOS, need for inotropic support, blood volume in drains): NS. Plasma PL levels of fatty acids: Preop: POAF group had lower levels of AA (*P <* 0.05) and higher levels of DHA (*P <* 0.05) compared with the non-POAF group. POD3: plasma PL levels of AA, EPA and total n-3 PUFA were lower than preop levels (*P <* 0.05), while those of DHA remained unchanged in both the non-POAF and POAF groups. The level of AA was still significantly lower, and the levels of EPA and DHA were higher in the POAF group compared with the non-POAF group: Lower levels of DHA and higher levels of AA in plasma PL were associated with decreased risk of POAF. PUFA may be beneficial for prevention of POAF in patients with very low baseline levels of total PUFA in plasma PL. AA may play an important electrophysiologic role.
**StR**: 2240 mg of n-3 PUFA daily PO (containing 1240 mg EPA & 1000 mg DHA) or 2000 mg olive oil for a week preop.	
**OM**: levels of fatty acids in phospholipids (PL) (preop & POD3), association between n-3 PUFA & n-6 PUFA in plasma PL and POAF (the incidence of POAF was compared between quartiles of the level of each fatty acid in plasma PL), Arachidonic acid (AA), CRP	
**Castillio**^[62]^, 2010, Chile	POAF: Intervention: 11/48–22.9% | Control: 15/47–31.9%, *P* = 0.32, Duration of AF (min): PUFA: 49 | Control: 15, *P* = 0.24. HLOS (d): PUFA: 9.20 ± 0.18 | Control: 8.15 ± 0.16, *P* = 0.02. Postop Complications: Intervention: 8.3% | Control: 10.6%, *P* = 0.51. AE: PUFA: 7 | Control: 6, *P* = 0.52. No further hemorrhagic or major adverse cardiovascular events (cardiovascular, non-fatal myocardial infarction or non-fatal stroke) were observed during 30 days of follow-up. Oxidative stress-related parameters: Lipid peroxidation (MDA) (lmol ⁄ mg protein): PUFA: 3.13 ± 0.83| Control: 4.32 ± 0.99. It was 27.5% lower in supplemented patients (*P* < 0.01). Protein carbonylation (nmol ⁄ mg protein): PUFA: 2.45 ± 0.75 | Control: 3.22 ± 0.75. It was 24% lower in supplemented patients (*P* < 0.01). GSH⁄GSSG ratio: PUFA: 10.53 ± 0.66 |Control: 7.63 ± 1.21. It was 38.1% higher in supplemented patients (*P* < 0.01). Inflammation-related parameters: Leucocyte count (cells ⁄ lL): PUFA: 7200 |Control: 6500, *P* = 0.02. Serum hs-CRP levels (mg ⁄ dL): PUFA: 2.02 | Control:1.56, *P* = 0.18. At discharge, supplemented patients showed leucocyte count and hs-CRP being 25.8 and 33.2% lower than placebo, respectively (*P* < 0.05). Atrial tissue DNA binding of NF-κB: in supplemented patients was 22.5% lower than that in placebo patients (*P* < 0.05). This study confirms that ischemia reperfusion induced by CS with CPB is associated with oxidative and inflammatory damage in atrial tissue. The combined n-3 PUFA-antioxidant vitamin protocol therapy reduced the oxidative stress and inflammation biomarkers, in patients undergoing on-pump CS.
**StR**: PO regimen of n-3 PUFA 2 g/day for 7 days preop, VitC 1 g/day & Vit E 400 IU/day from 2 days preop and all until discharge.	
**OM**: POAF, oxidative stress-related parameters (reduced ⁄ oxidized glutathione (GSH⁄GSSG) ratio, malondialdehyde (MDA), protein carbonylation, nuclear factor (NF)-kappaB activation, inflammation-related parameters: high-sensitivity C-reactive protein (hs-CRP), leucocyte count	
**Jackson**^[52]^, 2018, Italy	RBANS, MMSE, Trails A, and Trails B scores: it was observed an initial decline in cognitive performance followed by a recovery to baseline function by POD30. There were no statistically significant differences between groups (*P* = 0.32 | *P* = 0.57 | *P* = 0.40 | *P* = 0.42 respectively). Rates (%) of cognitive impairment by group across cognitive tests (day 0, day 4, day 30): wide variability in results was observed and no consistent patterns of impairment between groups were observed. Mean PUFA concentration: On the day of surgery in PUFA group increased by 38.59% (SS) but in control group essentially stayed the same. Perioperative supplementation with PUFA in CS patients did not influence cognition ≤30 d after discharge (both at days 4 and 30). NS differences were seen by treatment on a composite measure of neuropsychological functioning or individual cognitive measures over 30 d.
**StR**: Preop loading of 8–10 g PUFA (EPA 465 mg + DHA 375 mg) over 2–5 d & postop 2 g/d until hospital discharge or POD10 (whichever came first).	
**OM**: postop (over 30 d) cognitive decline in patients post CS through person assessments of cognition: RBANS, MMSE, Trails A and B tests	
**Masson**^[68]^, 2015, USA, Italy, Argentina	MACE: 8/562–1.5%. POAF: 3/173–1.7% | NOPOAF: 7/389–1.8%, *P* = 1. RBC transfusion: POAF: 74/173–40.8% | NOPOAF: 145/389–39.7 %, *P* = 0.5. 30-day mortality: 6/562–1.1%. Cardiac biomarkers (NT-proBNP & hs-cTnT): at the day of surgery: significantly higher in patients with POAF (*P* = 0.004, *P* = 0.002 respectively). Postop: both are markedly increased after CS with a different time course (NT-proBNP peaked on POD2 & hs-cTnT peaked at the end of surgery) but these changes were unaffected by fish oil supplementation (*P* > 0.05). Predictor of POAF: higher circulating levels of cardiac biomarkers preop as well as changes in their levels were not associated with risk of developing POAF (hs-cTnT adjusted HR = 0.84 [0.63–1.25], *P* = 0.49). & NT-proBNP adjusted HR = 0.89 [0.63–1.25], *P* = 0.49). PUFA treatment did not alter the time course of the cardiac biomarkers. NT-proBNP and hs-cTnT are related to clinical and surgical characteristics, have different perioperative time courses but are not independently associated with risk of POAF.
**StR**: Fish oil PO to a loading dose of 10 g divided over 3–5 days, including on the morning of surgery. Postop 2 g/day until hospital discharge or POD10 (whichever occurred first).	
**OM**: POAF, plasma concentrations of T-proBNP & hs-cTnT (preop, at the day of surgery, POD2), in-hospital MACE and 30-day mortality	
**Mariscalco**^[76]^, 2010, Italy	Early POAF: PUFA: 31% | Control: 47.3%, *P* = 0.006. OR = 0.54, 95% CI 0.31–0.92. Time of early AF occurrence (d): PUFA: 3.1 ± 1.7 | non-PUFA: 2.9 ± 2.2, *P* = 0.732. Recurrence rate: PUFA: 22.7% | non-PUFA: 25.9%, *P* = 0. 999. Persistence of the arrhythmia at hospital discharge: PUFA: 1.2% | non-PUFA: 7.8%, *P* = 0. 036. Late AF: PUFA: 11.9% | Control: 15.2%, *P* = 0.43, OR 1.0, 95% CI 0.49–2.21. Late AF was not influenced by preop PUFA regimen but a trend toward significance was observed in patients who received PUFA postop (*P* = 0.078). C-reactive protein (CRP, mg/L): PUFAs: 3.8 ± 2.9 | Control: 5.1 ± 4.0, *P* = 0 .025. White blood cell (WBC): early AF was associated with an increased level of WBC postop, with a significant peak on POD4 (*P* = 0.03). HLOS (d): POAF: 10.4 ± 9.8 | non-POAF: 9.5 ± 9.2, *P* = 0.025. Rehabilitation stay (d): POAF: 24.2 ± 15.3 | non-POAF: 21.1 ± 8.3, *P* = 0.008. Blood transfusion: POAF: 62% | non-POAF: 61%, *P* = 0.87. Preop PUFA therapy is associated with a 46% decreased incidence of early AF after CS but not late AF. Patients undergoing CS may benefit from a preventive PUFA approach.
**StR**: 1000 mg PO started on the day of operation & postop from POD1 for a median duration of 5 POD.	
**OM**: POAF during the surgical hospitalization (early AF) and/or the cardiac rehabilitation period (late AF).	
**Feguri**^[77]^, 2019, Brazil	POAF: PUFA: 10.3% | Control: 50%, RR = 4.83, 95% CI 1.56–15.02; *P* = 0.001, HLOS & ICULOS: NS differences (*P* = 0.713 & *P* = 0.980 respectively). Need for vasoactive drugs: after CPB: higher in control group (*P* = 0.071) | during recovery in the ICU: NS (*P* = 0.351). Amount of bleeding in the first 12 h of recovery in the ICU: NS (*P* = 0.351). Incidence of infectious complications in the ICU: NS (*P* = 0.179). Incidence of Combined Major Cardiovascular Events: NS (*P*>0.05). Blood Glucose Levels and Insulin Resistance: both groups receiving CHO had better glycemic control. The mean overall use of insulin in the ICU was significantly lower in groups that received CHO in comparison with the others (*P* = 0.018). Insulin resistance: blood glucose levels tend to increase during hypothermic CPB, whereas insulin levels tend to decline. Inflammatory Response – Inflammatory Markers: CRP: all patients presented a significant increase (*P <* 0.001) postop over time. W3 group (PUFA group) had lower levels of CRP within 36 h postop (*P* = 0.008). IL-6: NS differences (*P* = 0.105). IL-10: significant association between groups and serial tests (*P* = 0.013). PUFA (CHO + W3 and W3 groups) showed a significantly lower drop (*P* = 0.049) in mean values of postoperative recovery period (ICU, 6 h, 12 h, and 36 h), when compared to the groups that did not receive it. Fasting abbreviation with carbohydrate loading and intraoperative infusion of PUFA is safe and supports faster postop recovery in patients undergoing on-pump CABG because patients needed less vasoactive drugs during weaning from CPB or ICU recovery, lower incidence of POAF, improved metabolic control, and reduced postop inflammatory response.
**StR: Control group (n = 14**): 200 mL of water 2 h preop. **CHO group (n = 14**): PO 200 mL of water with 25 g (12.5%) of maltodextrin 2 h preop. **CHO + W3 group (n = 15**): 200 mL of water with 25 g (12.5%) of maltodextrin 2 h preop + intraop infusion of 0.2 g/kg of PUFA for 4 h. **W3 group (n = 14):** 200 mL of water 2 h preop + intraop infusion of 0.2 g/kg PUFA for 4 h.	
**OM**: POAF, infection, major combined CV events, need for vasoactive drugs, blood sugar level, assessment of insulin resistance, inflammatory response, bronchial aspiration during induction of anesthesia, bleeding in the first 12 h in the ICU, periop use of packed RBC and/or blood components, ICULOS, duration of mechanical ventilation, HLOS	
**Stanger**^[79]^, 2014, Austria	POAF: NS differences. Although there were indications for the shortest time for rhythm restoration in the Vit & PUFA subjects this was NS. Total peroxides: Substantial quantities of ROS emerged 6 hours after surgery until the end of observation, i.e. the third POD with significantly decrease in Vitamin/PUFA groups (groups 2&4). Although, the anti-oxidative effect of the vitamin/ PUFA supplementation was no longer active on POD2&3. Peroxidase activity: significantly reduced at the POD1 in groups 1, 2, 4 (*P* = 0.015 | *P* = 0.011| *P* < 0.01 respectively). In PUFA group there was no statistically significant difference in comparison to baseline values (*P* = 0.21). Significant decreases in peroxidase activities were observed only in groups 2 (*P* = 0.013) & 4 (*P* = 0.013) at POD2. oLAb: significantly decreased only in the control group (*P* = 0.002) at POD1. Water & Lipid-soluble antioxidants: significantly decreased at POD1&2 in all subgroups but recovered at POD3. Postop increase in oxidative stress was associated with the consumption of antioxidants and a simultaneous onset of AF. This was confirmed through an increased peroxide level and a decreased oLAb titer in control and PUFA groups. The administration of vitamins attenuates postop oxidative stress during CABG. Treatment with vitamins or PUFAs was inefficient with respect to AF onset and its duration. The discrepancy might be explained by the fact that patients with low baseline n-3 PUFAs levels may benefit disproportionately from DHA and EPA substitutions
**StR: Group 1**: Ctrl, **Group 2**: 500 mg VitC & 45IE VitE IV 30 min before reperfusion and 120 min after reperfusion. **Group 3:** 2 × 0.15 g fish oil/kg IV 42 h and 18 h preop and 50 mL 42 h postop. **Group 4**: 2 × 0.15 g fish oil/kg 42 h and 18 h preop and 50 mL 42 h postop IV plus 500 mg vitC & 45IE vitE IV 30 m before reperfusion and 120 m after reperfusion	
**OM**: POAF, Serum oxidative stress biomarkers: Total peroxides, Endogenous peroxidase activity, Antibodies against oxidized LDL (oLAb).	
**Mozaffarian**^[53]^, 2011, USA, Italy, Argentina	POAF: PUFA: 227/758–30% | Control: 233/758–30.7%, OR = 0.96, 95% CI = 0.77–1.20, *P* = 0.74. Secondary postoperative AF end points: NS. In-hospital MACE: PUFA: 1.7%| Control: 2.6%, *P* = 0.18. 30 days mortality: PUFA: 1.1%| Control: 2%, *P* = 0.14. Arterial thromboembolism (30d): PUFA: 0.7% | Control: 1.7%, *P* = 0.047, OR 0.37, CI 0.13–1.03. Arterial thromboembolism or death: PUFA: 1.7% | Control: 3.6%, *P* = 0.01, OR: 0.43, CI 0.22–0.84. ICULOS (d): PUFA: 2| Control: 2, *P* = 0.38. HLOS (d): PUFA: 7| Control: 7, *P* = 0.48. Total telemetry monitoring (d): PUFA: 6 | Control: 6, *P* = 0.39. Red blood cell transfusions: significantly fewer in PUFA group including during surgery (*P* = 0.002), postop (*P* = 0.008), and overall (*P <* 0.001). AE: minor, such as gastrointestinal upset, burping, and fish oil taste, occurred more commonly in the PUFA group. N-3-PUFA did not reduce the risk of POAF in CS, were generally well tolerated, with no evidence for increased risk of bleeding or serious AEs.
**StR**: preop loading dose of 8–10 g PO of n-3 PUFA or placebo divided over 2–5 days & postop 2 g/d until hospital discharge or POD10 (whichever came first).	
**OM**: POAF, major adverse cardiovascular events (MACE), 30-day mortality, bleeding, AE, ICULOS, HLOS	
**Gu**^[54]^, 2016, Denmark	POAF: 18/49–36.7%. POAF patients were older, with diabetes, more often females, had longer ECC-time, longer aortic cross-clamp time and longer postoperative ventilation time compared with NOPOAF patients. There were no correlations between the development of POAF and concentrations of n-3 PUFAs in atrial tissue and blood. Neither the content of total marine n-3 PUFA, EPA, and DHA in atrial tissue nor in plasma phospholipids predicted the development of POAF. Plasma phospholipids/right atrial tissue correlation (*r*): Significant correlations between the content of total n-3 PUFA, EPA, and DHA (but not DPA). EPA: *r* = 0.72 (strong), DHA: *r* = 0.52 (intermediate), DPA: *r* = 0.21 (no correlation), EPA + DHA: *r* = 0.60, EPA + DPA + DHA: *r* = 0.51. The study does not support a role for marine n-3 PUFA for the prevention of POAF, while it does not exclude a beneficial effect of supplemental n-3 PUFA in high(er) doses than obtained by seafood consumption alone. Measurement of n-3 PUFA in plasma phospholipids reflects – and is more convenient to obtain than – n-3 PUFA from the atrium.
**StR: –** Venous blood and tissue from the right atrial appendage were obtained from 50 patients undergoing elective CS.	
**OM**: the content of marine n-3 PUFA in atrial tissue and in plasma phospholipids was determined using gas chromatography.	
**Rodrigo**^[60]^, 2012, Chile	POAF: Patients <60 old: Intervention: 5/40–12.5% |Control: 12/40–30%, NS. Patients >60 years old: Intervention: 2/37–5.4% | Control: 10/35–2.8.5%, *P* = 0.0076. GSH-Px activity: at the day of the surgery, in atrial tissue was higher in patients >60 years who received the supplementation compared with the other groups (*P* < 0.05). Oxidative stress-related biomarkers and POAF: patients with POAF showed higher ↑plasma MDA levels postop (*P <* 0.0001) and lower ↓atrial GSH-px activity compared with those with no POAF (*P* < 0.01). The present study shows, for the first time, that older patients have a more marked antioxidant response to omega-3 plus vitamins C and E supplementation as a preventive treatment against POAF. Following supplementation, older patients had a 5 times lower risk of developing AF after CS, thus accounting for the greater effectiveness of the intervention in this group. Atrial GSH-Px activity seems to be an important predisposing factor for the development of POAF, as patients with higher activity showed lower incidence of this arrhythmia. Moreover, those patients who presented with postoperative AF also showed higher plasma MDA levels, a lipid peroxidation by-product. Higher enzymatic antioxidant activity (GSH-Px) and lower lipid peroxidation (MDA) biomarkers turned out to be correlated with lower POAF.
**StR**: PO regimen: 2 g/d n-3 PUFAs 7 days preop & Vit C (1 g/day) & Vit E (400 IU/day) 2 days preop and all continued until discharge.	
**OM**: POAF until hospital discharge, oxidative stress in plasma and atrial tissue samples: Malondialdehyde (MDA), Cu-Zn superoxide dismutase, catalase, glutathione peroxidase (GSH-Px) activity	
**Benedetto**^[63]^, 2011, Italy	Late mortality: lower risk in patients discharged on PUFA, HR 0.55; 95% CI, 0.26–0.90; *P* = 0.02. Patients with poor left ventricular function had better mortality benefit, HR 0.36; 95% CI, 0.17–0.76; *P* = 0.007. Repeat revascularization: significantly lower risk in PUFA group, HR 0.52; 95% CI, 0.28–0.97; *P* = 0.04. The adjusted risk for the composite of death, Q-wave myocardial infarction, or cerebrovascular events was lower in patients who received n-3 PUFAs compared with patients who did not (HR, 0.56; 95% CI, 0.36 to 0.81; *P* = 0.001). Other medications that showed a protective effect on overall mortality were aspirin (*P* = 0.002), b-blockers (*P* = 0.03), ACE inhibitors or angiotensin receptor blockers (*P* = 0.04), and statins (*P* = 0.001). Interestingly, the combination of clopidogrel plus aspirin did not add any benefit (*P* = 0.9). Authors support n-3 PUFA supplementation as part of standard medical therapy in all patients after CABG because n-3 PUFA supplementation is extremely safe and well tolerated, & there are currently few arguments against supplementation.
**StR**: 2 gelatin capsules (850 to 882 mg of EPA & DHA) after CABG starting at discharge from hospital. Median follow-up (d): PUFA: 908 | Control: 855	
**OM**: impact of n-3 PUFA supplementation on post-CABG outcomes: all-cause mortality, repeat revascularization, death, Q-wave myocardial infarction, cerebrovascular events.	
**Andreassen**^[45]^, 1997, Norway/USA	Blood pressure baseline: PUFA: 134/73 ± 5/3 mm Hg | Control: 126/70 ± 5/2 mm Hg (*P <* 0.05 for systolic blood pressure). Systolic blood pressure 6 months postop: PUFA: decreased 2 ± 4 mm Hg | Control: increased 17 ± 4 mm Hg, *P* < 0.01. Diastolic blood pressure 6 months postop: PUFA: increase 10 ± 3 mm Hg | Control: increase 21 ± 2 mm Hg, *P <* 0.01. Relation between systolic blood pressure and serum [PUFA]: *r* = −0.69, *P* = 0.01. Increase in 24 h hypertensive load from baseline to 6 months: PUFA: 60 ± 28 | Control: 149 ± 35 mm Hg, *P* < 0.05. The placebo group reveals a greater and more persistent increase in 24-h blood pressure after 6 months. Mean serum [PUFA]: increased significantly in the treatment group and remained unchanged in the placebo. Triglycerides: significantly reduced in the treatment group. Endothelial-dependent phase of the reactive hyperemic response: remained unchanged in the treatment group and decreased significantly in the placebo group. Time required for the hyperemic perfusion to return to pre-occlusive levels: increased slightly in the treatment group and decreased in the placebo group, *P*< 0.05. AE: gelatin capsules were well tolerated, and mild abdominal complaints were comparable between groups. Need for additional antihypertensive treatment at 6 months: PUFA: 5/14 | Control: 9/14. Postop daily administration of 4 g of PUFA in heart transplant recipients is effective as hypertension prophylaxis, depending on increases in serum EPA and DHA. Improved vascular reactivity in the treatment group, demonstrated by a more pronounced response to forearm skin ischemia than in the placebo group, indicates an endothelial protective effect that may contribute to the hypotensive role of n-3 PUFA.
**StR**: postop daily 4 g of PUFA PO started from POD4 until 6th month postop. In both active treatment and placebo capsules was added 3.7 mg of alpha-tocopherol as antioxidant. All the patients received a triple immunosuppressive regimen of cyclosporine, azathioprine and prednisolone.	
**OM**: Blood pressure, Skin-reactive hyperemia (represent microvascular endothelium-dependent vasodilation), Laboratory values.	
**Akintoye**^[49]^, 2016, USA, Italy, Argentina	Levels of all MEFAs declined substantially following surgery (at POD2), with declines ranging from 37% to 63% (*P* < 0.05 each). Compared with placebo at POD2, levels of EPA- and DHA-derived MEFAs were 40% and 18% higher, respectively (*P* ≤0.004). Both enrollment level and changes in plasma phospholipid EPA and DHA were associated with their respective MEFAs at POD2 (*P* < 0.001). N-3 PUFA supplementation significantly ameliorated the reduction in postop n-3 MEFAs, but not n-6 MEFAs, and the degree of increase in n-3 MEFAs related positively to the circulating level of their n-3 PUFA precursors (from enrollment to the morning of CS). The availability of dietary-derived n-3 PUFA substrate in phospholipids contributes to the in vivo regulation of MEFA concentrations.
**StR**: 8–10 g PUFA PO (1gr = 465 mg of EPA + 375 mg) for 2–5 days preop & 2 g/d postop until discharge/POD 10 (whichever came first) or placebo (olive oil).	
**OM**: dynamics of monoepoxides derived from polyunsaturated fatty acids (MEFAs) and their response to n-3 PUFA suppl.	
**Charman**^[64]^, 2005, UK	WBC (at recruitment, preop, at rewarming): NS between-group differences. Fish oil did not significantly decrease post-CPB neutrophil activation (as detected with ex vivo MPO, *P* = 0.026, & SAG, *P* = 0.012) but may moderate postop myocardial damage. Neutrophil apoptosis: At recruitment: PUFA: 24.6 ± 11.0% | Control: 30.2 ± 14.0%, *P* = 0.8. At rewarming: PUFA: 12.9 ± 3.5%| Control: 15.4 ± 6.7% (*P* = 0.7). Neutrophil apoptosis: apoptosis at end-CPB was equally reduced in both groups: PUFA: from 23 ± 9% to 13 ± 4% (*P* < 0.001) | Control: 35 ± 14% to 15 ± 3% (*P* = 0.001). Post-operative blood loss: At 6 h: PUFA: 738 ± 414 mL | Control: 549 ± 458 mL, *P* = 0.2. At 12 h: PUFA: 1123 ± 1141 mL| Control: 1190 ± 506 mL, *P* = 0.14. One patient in the placebo group underwent resternotomy for bleeding. Blood Lipids: At recruitment was seen significantly lower levels of HDL cholesterol at PUFA group. Postop: PUFA group: plasma VLDL: ↓decreased from 0.69 ± 0.34 to 0.51 ± 0.24 mmol/L (*P* = 0.007), Triglycerides: decreased from 1.68 ± 0.70 to 1.39 ± 0.54 mmol/L (*P* = 0.02), HDL cholesterol: increased (*P* = 0.0003). Control group: decreased HDL cholesterol and increased triglycerides & VLDL. Troponin I: At end-CPB overall troponin I levels averaged 0.91 ± 0.60 ng/mL which clearly exceeded diagnostic levels (0.15 ng/mL). At 24 h troponin I fell significantly in the fish oil group to 46 ± 23% of end-CPB levels (*P* = 0.0002) whereas it peaked in the placebo group to 107 ± 72% (*P* = 0.013); This study detected a small anti-neutrophil effect, no significant increases in bleeding attributable to fish oil, and decreased post-operative release of troponin I which may be an important protective effect.
**StR**: 8 g/day fish oil PO or placebo for 6 weeks.	
**OM**: neutrophil behavior under the influence of dietary fish oil: neutrophil activation: superoxide anion generation (SAG), myeloperoxidase release (MPO), neutrophil apoptosis, cardiac damage, end-organ damage.	
**Rubanenko**^[44]^, 2022, Russia	POAF: PUFA: 16.9% | Control: 29.7%, *P* = 0.009. The association with POAF became statistically insignificant (*P*>0.05) for postop MDA hemoglobin concentration >0.78 μmol/g. POAF predictors (multivariate regression): Postop hemoglobin RG concentration ≤0.194 μmol/g: OR = 4.0; 95% CI 1.1–14.3, *P* = 0.030. Postop IL-6 after surgery >19.53 pg/mL: 4.4; 95% CI 1.3–15.4, *P* = 0.020. Postop omega-3 index ≥1.83%: 0.4; 95% CI = 0.3–0.54, *P <* 0.001. Postop plasma SOD >1129.6 U/g: 4.5; 95% CI 1.2–17.8, *P* = 0.040. PUFA context changes postop compared with preop: EPA: PUFA: ↑33.3% (*P* = 0.29) |Control: ↓25% (*P* = 0.140), DHA: PUFA: ↑31.8% (*P* = 0.010) |Control: ↓47.9% (*P* = 0.080), Omega-3 index: PUFA: ↑44.3% (*P* = 0.010) |Control: ↓26.3% (*P* = 0.020). Postop the DHA concentration in PUFA group was 55% higher (*P* = 0.030), and the omega-3 index was 43.4% higher (*P* = 0.040) compared with control. Inflammatory parameters change postop: Control vs PUFA group: IL-6 level: ↑83.4% (*P <* 0.001) | ↑74% (*P <* 0.001). IL-8: ↑78.2% (*P <* 0.001) | ↑ 68.2% (*P <* 0.001). IL-10: ↑48.3% (*P* < 0.001) | ↑ 42.3% (*P <* 0.001). NT-proBNP: ↑74.1% (*P* < 0.001) | ↑ 72% (*P* < 0.001). Plasma SOD activity: ↓30% (*P* < 0.001) | ↓ 82.8% (*P* < 0.001). Erythrocyte glutathione reductase activity: ↑10.2% (*P* = 0.040) | ↑ 11.3% (*P* = 0.040). Erythrocyte RG level: ↓21.4% (*P* = 0.002) |↓32% (*P* = 0.003). MDA: ↑32% (*P* = 0.001) | ↑ 19% (*P* = 0.030). Thus, a lower activation of the inflammation↓ and oxidative stress↓ parameters was observed against the background of an increase in the DHA↑ level and the omega-3 index↑ among patients taking PUFA after CABG, which was accompanied by a decrease in the incidence of AF↓.
**StR:-Group 1:** No PUFAs (Controls)**. Group 2:** PO, 2000 mg 5 days preop & and 1000 mg postop for 21 days (Intervention)	
**OM**: POAF. All patients underwent a study of the content of interleukins: 6 (IL-6), 8 (IL-8) and 10 (IL-10). The concentrations of NT-proBNP, troponin, plasma superoxide dismutase (SOD), malondialdehyde (MDA), reduced glutathione (RG) and the activity of glutathione reductase (GR) enzymes, omega-3 index were also investigated.	
**Berger**^[56]^, Switzerland, 2012	Membrane composition and plasma lipids: PUFAs increased EPA & DHA concentrations in platelets and the EPA concentration in atrial tissue within 12 h of the first fish oil administration. Effects on postop systemic inflammation: IL6 (*P* = 0.018) & IL8 (*P* = 0.005) significantly decreased in PUFA group. CRP on POD1 was not significantly different among groups. Carboxyhemoglobin the first 24 h postop was significantly lower in PUFA group. Effects on organ function and major clinical outcomes: NS differences for arrhythmias. The mean SOFA score and its change over time did not differ significantly between groups. The length of mechanical ventilation tended to be 11 h shorter in the PUFA group (*P* = 0.10). ICULOS was 16 h shorter in PUFA group (*P* = 0.118). APACHE II was lower in PUFA group (*P* = 0.058). Heart rates were modestly higher in the FO group during the first 12 h postop. Effects on glucose metabolism: average glycemia value during the first 24 h after CPB was significantly lower in PUFA group. Insulin requirements during the first 24 h did not differ significantly. Plasma lactate was lower in PUFA group (*P* = 0.0077). No adverse effect of PUFA was detected. The results underline the importance of cell membrane incorporation of n-3 PUFAs in inflammatory and cardiac cells to achieve relevant biological effects. This incorporation is more rapid than previously known and comes with biological and clinically relevant changes during the early postop course characterized by attenuated inflammatory and blunted metabolic responses. These results suggest that perioperative FO may be beneficial in elective CS.
**StR**: PUFA (0.2 g/kg) IV infusions 12 & 2 hours preop and immediately postop or saline solution (control).	
**OM**: membrane composition of atrial tissue and circulating platelets, modulation of the inflammatory response, glucose metabolism, cardiovascular status, postop clinical course.	
**Eritsland**^[55]^, Norway, 1995	40.8% of the patients in the PUFA group and 47.4% in the ctrl group received ASA (*P* = 0.13); the others warfarin. The amount of serum n-3 PUFA ↑ 45% in the fish-oil group and was nearly unchanged in the ctrl group. More pronounced for ↑ EPA (140%), but also significant ↑ for DHA (14%). The n-6 fatty acids ↓ by 7% in the fish-oil group and ↑ by 10% in the control group. There were no significant differences in the frequency of bleeding episodes between patients in the fish-oil and the control groups (*P* = 0.53), neither between patients given aspirin and those given warfarin (*P* = 0.28). The platelet count ↑in both groups during the study period, but less so in the fish-oil group (*P* = 0.048). No group differences were noted in bleeding time (*P* = 0.27) or in plasma beta-thromboglobulin (BTG) levels (*P* = 0.87). Serum triglyceride (TG) levels ↓ by 19.1% in the fish-oil group and were unchanged in the ctrl group. No group differences in the levels of fibrinogen, FYI’, TAT, FPA, D-dimer, or PAI-1 activity were noted. With the moderate PUFA dose supplied in the present study, no significant long-term influence by n-3 PUFAs on the measured hemostatic variables was seen. Furthermore, no excess of bleeding episodes could be attributed to the use of fish-oil concentrate, even when given in addition to aspirin or warfarin. A beneficial influence of n-3 PUFAs on thrombotic complications, however, is not excluded by the present observations, as potential antithrombotic effects exerted locally at the cellular level in the vessel may occur.
**StR**: PUFA group, PO, 4 g fish-oil concentrate (51% EPA, 32% DHA, 14.8 mg VitE) randomized on the POD2 vs ctrl (non-supplemented) group. Simultaneously, randomized in a factorial 2 × 2 design to either aspirin (ASA) 300 mg/day or warfarin aiming international normalized ratio (INR) 2.5-4.2. Duration 9 months.	
**OM**: Long-term effects of a moderate dose of *n-3* PUFAs, supplied as a fish-oil concentrate, on hemostatic variables (platelets, coagulation and fibrinolysis), all bleeding episodes were recorded.

**Table 6:** Abbreviations and acronyms used in the review.

6moPO	6 months postoperatively
AAD	Antiarrhythmic drug
ACE	Angiotensin-converting enzyme
ADHD	Attention deficit hyperactivity disorder
ADP	Adenosine diphosphate
AE	Adverse events
AF	Atrial fibrillation
AHA	American Heart Association
ALA	α-Linolenic acid
ANOVA	Analysis of variance
APACHE II	Acute physiology and chronic health evaluation
ASPI	Arachidonic acid test
BARC	Bleeding Academic Research Consortium
BNP	Brain natriuretic peptide
CABG	Coronary artery bypass graft surgery
CAD	Coronary artery disease
CHD	Coronary heart disease
CI	Confidence interval
CK-MB	Creatine kinase – myocardial band
COL	Collagen
CPB	cardiopulmonary bypass
CS	cardiac surgery
Ctrl	Control
D	Days
DB	Double blinding
DHA	Docosahexaenoic acid
DPA	Docosapentaenoic acid
EC	Exclusion criteria
EF	Ejection fraction
eGFR	Estimated glomerular filtration rate
EPA	Eicosapentaenoic acid
FA	Fatty acids
FDA	Food and Drug Administration
FMD	Flow-mediated dilation
GR	Glutathione reductase
GRAS	Generally recognized as safe
GSH-px	Glutathione peroxidase
H	Hours
HDL	High-density lipoprotein
HF	Heart failure
HLOS	Hospital length of stay
HRV	Heart rate variability
hs-CRP	High-sensitivity C reactive protein
hs-cTnT	High-sensitivity cardiac troponin T
I	Intervention group
IBM	International Business Machines
I/R	Ischemia/reperfusion
IC	Inclusion criteria
ICU	Intensive care unit
ICULOS	Intensive care unit length of stay
IL6/8	Interleukin 6/8
IV	Intravenous
LDL	Low-density lipoprotein
LV	Left ventricle
LVEF	Left ventricle ejection fraction
M	Months
MACE	Major adverse cardiovascular events
MDA	Malondialdehyde
MEFAs	Monoepoxides derived from polyunsaturated fatty acids
MHC II	Major histocompatibility complex II
MI	Myocardial ischemia/infraction
N-3 PUFAs	Polyunsaturated fatty acids
NB	Non-blinding
ND	Non-defined
NO	Nitric oxide
NOPOAF	Non-postoperative atrial fibrillation
NS	Non-statistically significant
NT-proBNP	N-terminal pro-B-type natriuretic peptide
NYHA	New Work Heart Association
oLAb	Antibodies against oxidized LDL
OM	Outcome measures
OP	Operation
OR	Odds ratio
P.o.	Oral administration
PDGF	Platelet derived growth factor
PGI2	Prostaglandin I2
PL	Phospholipids
POAF	Postoperative atrial fibrillation
POD	Postoperative day
Postop	Postoperative/postoperatively
Preop	Preoperative/preoperatively
pro-ANP	Proatrium natriuretic peptide
Prosp	Prospective
PUFA(s)	Polyunsaturated fatty acid(s)
RBC	Red blood cells
RCT(s)	Randomized control trial(s)
RG	Reduced glutathione
ROS	Reactive oxygen species
SAG	Superoxide anion generation
SD	Standard deviation
SDA	Stearidonic acid
SOD	Copper (Cu)-Zinc (Zn) superoxide dismutase
SPSS	Statistical Package for the Social Sciences
SR	Sinus rhythm
SS	Statistically significant
StR	Study regimen
TB	Triple blinding
TG	Triglycerides
TGF-β	Tumor Growth Factor-beta
TIMI	Thrombolysis in Myocardial Infarction
TnT	Troponin I
TRAP	Thrombin receptor-activating peptide
Vit	Vitamin
VLDL	Very low-density lipoprotein
WBC	White Blood Cells

### Dosing, timing, duration, and administration routes of n-3 PUFA supplementation

The dosing regimen used in the identified studies was generally heterogeneous, more frequently orally (PO) administered and in a few occasions intravenously (IV). Many studies used 2 g of fish oil for 2–5 days pre-operatively. Post-operatively, usually 1–2 g/day or less of fish oil was administered for some days, most frequently for up to 5–10 days, or until hospital discharge. However, dosage as high as 4.6 g/d, have also been reported.^[48]^ To our knowledge, there is no dose-finding study on CS patients yet. FDA has advised that adults can safely consume up to a total of 3 g/day of combined DHA and EPA, with no more than 2 g from dietary supplements^[59]^.

In most of the studies, n-3 PUFA were administered alone, whereas in a few other studies they were administered alongside with other antioxidants, such as vitamin C and vitamin E. The concomitant administration in these studies varied depending on the study design and the clinical endpoints under investigation.

Nonetheless, the majority of studies exclusively investigated the effects of n-3 PUFA. In these studies, n-3 PUFA were administered pre-operatively in the same (or in somewhat higher) doses and for shorter periods than post-operatively. Interestingly, the most common regimen was 2 g orally for 2–5 days before surgery, which was followed by 1–2 g daily for 5–10 days post-operatively, usually until hospital discharge; and the same applied for combined regimens (vitamins C & E & n-3 PUFA).^[60-62]^ Variations in dose or in its daily dose division occurred amongst different authors. Seemingly, only a handful of authors have examined the effects of a longer-term supplementation in this group of patients, namely beyond hospital discharge^[44,45,55,63,64]^. Pre-operative administration regimes varied among authors especially among those administering combined therapies and followed no special pattern. Some authors administered a preoperative loading dose of 8–10 gr fish oil divided in 5 days.^[50,52,65-68]^ All in all, we identified no patterns, nor any dose-response relationships. We thereby assume that beyond the inherent heterogeneity bias in our study, n-3 PUFA incorporate rapidly in body’s cell membranes^[69-73]^, reaching a saturation level, where the excessive bioavailable lipid soluble n-3 PUFA^[3,74]^ has seemingly no additional effect^[75]^.

## Discussion

Though not consistent in all authors, there seems to be a beneficial effect of n-3 PUFA supplementation in postoperative arrhythmias such as in POAF,^[11,44,60,61,65-67,76,77]^ reduction of ICULOS^[11,48,56,67]^, and HLOS^[11,61,62,65,67,76]^, reduction in post-operative ventilation time^[56]^, in arterial thromboembolism^[53]^, need for less vasoactive drugs after CPB^[77]^, faster postop recovery^[77]^, lower risk for repeat revascularization^[63]^, improved mortality^[53,63]^, and quite interestingly less postop fatigue^[78]^. Rodrigo *et al* even suggested that n-3 PUFAs combined with vitamins C and E resulted in a more marked reduction of POAF in older patients, thus suggesting, that the efficacy of this therapy improves with aging, and that older patients have a more efficient antioxidant response to N-3 PUFA. Following supplementation, older patients had a 5 times lower risk of developing AF after CS^[60]^. Moreover, Skuladottir *et al* suggested that PUFA may be beneficial for prevention of POAF in patients with very low baseline levels of total PUFA in plasma PL^[58]^, a theory that is also supported from Stanger *et al*^[79]^ and Rubanenko *et al*^[44]^, while Mariscalco *et al*^[76]^ proved that preop PUFA therapy is associated with a 46% decreased incidence of early AF after CS but not late AF. Moreover n-3 PUFA increased antioxidant potential^[60]^, and attenuated oxidative stress^[61,62,79]^, and inflammation^[44,56,61,62,66,77]^, with subsequent decrease of serum TnT^[64,80]^, lactate^[56]^, glycemia^[56]^, plasma MDA levels (a lipid peroxidation by-product)^[44,60,62]^, CK-MB^[80]^ and CRP levels^[76,77]^, ROS peroxides^[79]^, triglycerides^[45,55,64]^, very low-density lipoprotein (VLDL)^[64]^, antibodies against oxidized LDL (oLAb)^[79]^, IL6-8-10 levels^[44,56]^, NT-proBNP^[44]^, carboxyhemoglobin^[56]^, preservation of white blood cell^[62,64,76]^ and peroxidase activity^[79]^. Furthermore, they increase HDL^[64]^, GSH⁄GSSG ratio^[62]^, the activity of catalase, superoxide dismutase (SOD)^[44]^, and glutathione peroxidase (GSH-Px)^[60]^ in atrial tissue of the supplemented patients^[61]^, thus promoting the extraction of oxygen and lactate uptake in the PUFA group^[80]^ with significant reduction in myocardial ischemic-reperfusion injury^[62,80]^, and early metabolic recovery of the heart after elective CABG, earlier shift to aerobic metabolism during reperfusion and improved myocardial protection^[80]^, thus moderating postop myocardial damage^[64]^. Moreover, Andreassen *et al* suggest that PUFA improves vascular reactivity in heart transplant recipients and is effective as hypertension prophylaxis, depending on increases in serum EPA and DHA, suggesting an endothelial protective effect that may contribute to the hypotensive role of n-3 PUFA^[45]^. These results underline the importance of cell membrane incorporation of n-3 PUFAs in inflammatory and cardiac cells to achieve relevant biological effects^[56]^.

There have been quite controversial results, mostly non-significant, about the potential beneficial effects of n-3 PUFA in other organs/systems (Table 1) and in the occurrence of various AEs (Table 2), such as postop blood loss, major bleeding, red blood cell transfusions, and frequency of re-operation. The scientific literature, as seen in Tables 1 and 2, clearly supports all these beneficial or adverse effects, but the results of the studies included in our review have not always supported the case, and although they are indicative, still they are not conclusive. Beneficial influences of n-3 PUFAs on thrombotic complications, are not excluded by the present studies’ observations as potential antithrombotic effects exerted locally at the cellular level in the vessel wall occurs^[55]^, and may be of importance^[81]^. The activity of platelets was lower postop, particularly in the collagen (COL) test in the PUFA group, but seemingly with no negative effect on bleeding^[82]^. Lastly, a number of combination studies that included n-3 PUFA resulted in various promising findings. It is worth emphasizing that there is a consensus in the results of these authors regarding the ability of n-3 PUFA in combination with other antioxidants to attenuate oxidative stress in cardiac post-operative patients.^[60**^-^**62,78,79]^ Their findings might help identify the patients who are expected to be benefited most from pre-operative n-3 PUFA plus antioxidant vitamins supplementation and consequently, establish the criteria to choose which patients will receive this prophylactic treatment^[60]^. Moreover, Gholami *et al*^[78]^ presented that the combination of vitamin C and n-3 PUFA can effectively reduce the level of a majority of fatigue dimensions including general and physical fatigue, as well as reduced activity and motivation to live among patients following a CABG. This could be to the synergic effects of vitamin C and n-3 PUFA on reducing pro-inflammatory mediators as well as the effect of vitamin C as a cofactor on transferring fatty acids into the cells and generate energy^[78]^.

Finally, we tried to compare our results with other relevant reviews. In the systematic review and meta-analysis from Langlois *et al*^[83]^ including 19 RCT with 4335 patients, no effect of n-3 PUFA on mortality, mechanical ventilation (MV) duration or ICULOS was found. However, n-3 PUFA helped to reduce hospital LOS and POAF incidence. Nonetheless considerable clinical and statistical heterogeneity, which even remained in subgroup analysis, weakened their findings. In another meta-analysis by Zhang *et al*, with eight RCT trials and 2687 patients, authors suggests that treatment with PUFA preoperatively has no effect on the incidence of POAF in patients undergoing open heart surgery; and that diabetics might benefit from PUFA treatment preoperatively^[84]^. Yet again, results obtained from this study are inconclusive, likely attributed to the small number of trials and patients included.

### Adverse health effects

N-3 PUFA are generally well tolerated, however excessive intake or imbalanced consumption can lead to adverse effects, primarily associated with high doses from supplements rather than dietary sources. Firstly, due to their ability to inhibit platelet aggregation, high doses of n-3 PUFA can reportedly increase the risk of bleeding.^[85-87]^ In our review, this consensus is contradicted by many authors^[47,55,58,62,64,77,82]^ who suggest that non-significant differences exist between controls and n-3 PUFA group in bleeding or red blood cell transfusions, while others even showed that fish oil supplementation decreased perioperative bleeding and reduced the number of blood transfusions^[48,53,57]^. Quite interestingly in these studies, higher achieved n-3-PUFA levels were associated with lower risk of bleeding. These novel findings support the need to reconsider current recommendations to stop fish oil or delay procedures before CS^[57]^. Eritsland *et al*, found no significant long-term influence of n-3 PUFA on various measured hemostatic indices examined. Furthermore, no excess of bleeding episodes could be attributed to the use of fish-oil concentrate, even when given in addition to aspirin or warfarin^[55]^. Secondly, high consumption of n-3 PUFA may cause gastrointestinal discomfort^[47]^, including diarrhea, bloating, and belching. In addition, excessive intake of n-3 PUFAs can disrupt the balance between omega-3 and omega-6 fatty acids, potentially increasing the risk of chronic inflammation-related diseases^[88]^. Despite their known anti-inflammatory properties, some studies suggest that high intakes of n-3 PUFA could potentially suppress the overall immune body’s function^[89]^. N-3 PUFA may interact with some medications altering their effects. These drugs include anticoagulants, antiplatelets, beta-blockers, diuretics, estrogen-based contraceptives, and hormone replacement therapy^[90]^. Allergic reactions or hypersensitivity to fish and shellfish are known contraindications for taking n-3 PUFA^[6]^. In our review, out of 4991 patients that received n-3 PUFA only a single case of allergic response was reported^[65]^. PUFA treatment was proven to be well tolerated without serious AEs compared with placebo^[47,48,51,53,56,65,77,91]^. Considering these data, the AHA in 2006 came up with a consensus statement on secondary prevention for lipid management, that encourages increased consumption of n-3 PUFA in the form of fish or in capsule form (1 g/day) for risk reduction^[92]^. Some authors even support n-3 PUFA supplementation as part of standard medical therapy in all patients after CABG for reducing cardiovascular risk and mortality^[11,93]^, arguing that n-3 PUFA supplementation is extremely safe^[46]^, and that there are currently few arguments against supplementation^[63]^.

### Study limitations

By design, our narrative literature review lacks homogeneity and is not systematic in covering all possible studies, nor a relevant meta-analysis is conducted due to lack of adequate power. Unavoidably, there are population, intervention, study design and outcome measures differences that confer largely to bias and heterogeneity. Key confounding variables include patient-related factors such as gender, age, baseline nutritional status, other medication used and concurrent antioxidant therapy, EPA:DHA ratio, comorbidities, different types of surgery and surgical techniques, dose, timing, duration and route of n-3 PUFA administration; lack of comprehensive formal quality assessment of the trials included, etc. Due to these inconsistencies and heterogeneity in the studies included, it is not feasible to draw conclusions or statistical inference on how dosing, duration, and administration routes of n-3 PUFA supplementation differences impact the results. While a narrative review cannot utilize statistical methods to control for confounders as systematic reviews and meta-analyses can, we acknowledge that these variables are critical and likely impact the interpretation of our findings. The heterogeneity suggests that the optimal dose, patient population, and duration of n-3 PUFA administration to improve clinical outcomes remain unclear. Although narrative reviews inherently lack the ability to clarify or investigate heterogeneity and it is prone to selection and other types of bias, we have made every effort to remain objective with the state of the science related to the problem at hand. A future, adequately powered, systematic review of RCTs and a meta-analysis could definitely address more comprehensively the postoperative administration of n-3 PUFA in CS patients.

## Conclusion

In summary, we identified several prospective cohort studies and RCTs which show that n-3 PUFA is a safe and affordable supplementation therapy that may help in various patients who are undergoing CS or other cardiac procedures. N-3 PUFA reduce the risk of significant cardiovascular complications and costs, both peri- and postoperatively. The latter is particularly beneficial because remodeling and restoration of the heart’s muscle and nerve tissue occur during the same time period.

Yet, several research questions remain under consideration. Should n-3 PUFA be administered routinely in CS patients? Have n-3 PUFA a place in CS peri- and postoperative optimal therapy? Which patient population could benefit most from this regimen? For how long, in which dose and by which route should it be administered? Should n-3 PUFA be administered solely or in combination with other antioxidants and/or other medications? Future investigations need to address such scientific questions through more sophisticated RTCs, preferably organized as multi-center studies, adequately powered, well stratified, and blinded, in order to enlighten the present knowledge gap about the post-operative use of n-3 PUFA, following CS. Considering the current evidence, one can hypothesize, that n-3 PUFA supplementation for a longer period might benefit CS patients in ways that have not been previously reported in the scientific literature. This potentially paves the way for improvements in the outcomes of CS patients as well as in- and outpatient cost effectiveness of n-3 PUFA administration in CS.

## Data Availability

Not applicable.
